# Quantifying dose-, strain-, and tissue-specific kinetics of parainfluenza virus infection

**DOI:** 10.1371/journal.pcbi.1009299

**Published:** 2021-08-12

**Authors:** Lubna Pinky, Crystal W. Burke, Charles J. Russell, Amber M. Smith

**Affiliations:** 1 Department of Pediatrics, University of Tennessee Health Science Center, Memphis, Tennessee, United States of America; 2 United States Army Medical Research Institute for Infectious Diseases, Fort Detrick, Maryland, United States of America; 3 Department of Infectious Diseases, St. Jude Children’s Research Hospital, Memphis, Tennessee, United States of America; University of Pittsburgh, UNITED STATES

## Abstract

Human parainfluenza viruses (HPIVs) are a leading cause of acute respiratory infection hospitalization in children, yet little is known about how dose, strain, tissue tropism, and individual heterogeneity affects the processes driving growth and clearance kinetics. Longitudinal measurements are possible by using reporter Sendai viruses, the murine counterpart of HPIV 1, that express luciferase, where the insertion location yields a wild-type (rSeV-luc(M-F*)) or attenuated (rSeV-luc(P-M)) phenotype. Bioluminescence from individual animals suggests that there is a rapid increase in expression followed by a peak, biphasic clearance, and resolution. However, these kinetics vary between individuals and with dose, strain, and whether the infection was initiated in the upper and/or lower respiratory tract. To quantify the differences, we translated the bioluminescence measurements from the nasopharynx, trachea, and lung into viral loads and used a mathematical model together a nonlinear mixed effects approach to define the mechanisms distinguishing each scenario. The results confirmed a higher rate of virus production with the rSeV-luc(M-F*) virus compared to its attenuated counterpart, and suggested that low doses result in disproportionately fewer infected cells. The analyses indicated faster infectivity and infected cell clearance rates in the lung and that higher viral doses, and concomitantly higher infected cell numbers, resulted in more rapid clearance. This parameter was also highly variable amongst individuals, which was particularly evident during infection in the lung. These critical differences provide important insight into distinct HPIV dynamics, and show how bioluminescence data can be combined with quantitative analyses to dissect host-, virus-, and dose-dependent effects.

## Author summary

Human parainfluenza viruses (HPIVs) cause acute respiratory infections and can lead to the hospitalization of children. HPIV infection severity may vary due to dose, strain, patient, and whether the infection initiates within the upper or lower respiratory tract. There is a need to determine how the rates of virus spread and clearance change in different infection scenarios in order to better understand varying clinical manifestations. The significance of our research is in identifying the dominant mechanisms driving strain-, dose-, and tissue-specific HPIV infection kinetics, and in pairing bioluminescence data with quantitative analyses to determine how the same virus can yield patient-specific outcomes. This work enhances our understanding of HPIV infection and broadens our knowledge viral dynamics in the upper and lower respiratory tracts.

## Introduction

Human parainfluenza viruses (HPIVs) are a leading cause of acute respiratory infection, with 80% of children seropositive by the age 5 [1]. As a consequence, pediatric hospitalization rates are second only to respiratory syncytial virus (RSV) [2]. In healthy young adults, this illness is typically mild, self-limited, and restricted to the upper respiratory tract. However, in infants and young children, HPIVs cause lower respiratory tract infections that can result in severe illnesses such as croup, bronchiolitis, and pneumonia [3, 4]. Further, immunity following an infection may be short-lived making individuals susceptible to re-infection [5]. The factors influencing disease outcome are understudied [6] and there are conflicting reports about whether serotype-specific kinetics and host responses result in differing clinical manifestations [7]. With no vaccines or antivirals approved to treat HPIV infection, a better understanding of the infection kinetics and how these may change with different doses, viral attenuation, and infection site is vital to effectively abrogating HPIV-related illnesses.

The murine parainfluenza counterpart, Sendai virus (SeV) [8], has been used extensively to understand HPIV infection [9–13]. SeV infection can be studied using luciferase reporter SeVs [14–16] enabling noninvasive bioluminescence imaging to study the temporal and spatial dynamics in the respiratory tracts of living animals during HPIV infection [14–16]. Luciferase insertion at the M and F junction (rSeV-luc(M-F*)) yielded a wild-type-like virus while insertion at the P and M gene junction (rSeV-luc(P-M)) resulted in a virus with an attenuated phenotype [14]. Animals infected with a high dose in a high volume (“high d/v”; 7000 plaque forming units (PFU) in 30μl) of either virus lead to viral growth throughout the respiratory tracts and caused high level of lung infection with severe disease condition in the mice [16]. Conversely, animals infected with a low dose in a low volume (“low d/v”; 70 PFU in 5μl) initiated an upper respiratory tract (URT; nasopharynx) infection that ultimately migrated to the lower respiratory tract (LRT; trachea and lung) after ∼2 d with reduced dissemination in the lung [16]. Infection at the low d/v with either virus resembled infection dynamics after contact transmission [16]. These data showed that different doses and strains initiated distinct levels of infection in the nasopharynx, trachea, and lung. Understanding which infection processes drive the differing dynamics may provide insight into the pathogenicity and transmissibility of HPIV infection.

Mathematical models are useful to understand and quantify *in vivo* kinetics of a myriad of viral infections and have been used to analyze other respiratory viruses like influenza A virus (IAV) (reviewed in [6, 17–19]), respiratory syncytial virus (RSV) [20–22] and severe acute respiratory syndrome coronavirus 2 (SARS-CoV-2) [23–26]. A strength of these models is that they can estimate the rates of infection that are not easily measured within the laboratory or clinic (e.g., virus production and infected cell half-life) and define the primary infection processes that drive differing kinetics (e.g., between strains or doses; e.g., as in [27, 28]) in addition to their magnitude. To date, no modeling study has assessed the *in vivo* dynamics of HPIVs, which is the focus of this work.

Here, we pair a mathematical model [29] together with bioluminescence data from mice [16] to identify how viral attenuation, dose, respiratory tissue, and individual heterogeneity affect the kinetic rates of parainfluenza virus infection. The analyses confirmed that the attenuated strain has a lower rate of virus production compared to the wild-type-like strain, and that low dose infections result in disproportionately fewer infected cells compared to high dose infections. In addition, the rates of infectivity and infected cell clearance were highest in the lung and slowest in the nasopharynx. In the LRT, this rate was distinct between individuals with increased values in high d/v infections. Together, these analyses highlight the underlying processes that drive distinct kinetics resulting from different infection scenarios.

## Materials and methods

### Experimental data

The data used are from Burke et al. [16]. Briefly, groups of 15 129X1 mice were intranasally infected with rSeV-luc(M-F*) or rSeV-luc(P-M) at a low dose/volume (“d/v”) (70 PFU in 5 μl) or high d/v (7000 PFU in 30 μl). Bioluminescence (the total flux (photons/second)) in the nasopharynx, trachea, and lungs was measured daily for 10 d in all mice. To obtain the relation between bioluminescence and viral loads, a separate experiment was completed where groups of 5 mice intranasally inoculated with a low d/v or high d/v of rSeV-luc(M-F*) or rSeV-luc(P-M) were noninvasively imaged to obtain bioluminescence after 3, 5, or 7 days of infection before nasal, tracheal, and lung tissues were harvested from the same animal so that viral loads could be measured by plaque titration.

### Translating bioluminescence to viral load

**Fig 1:**
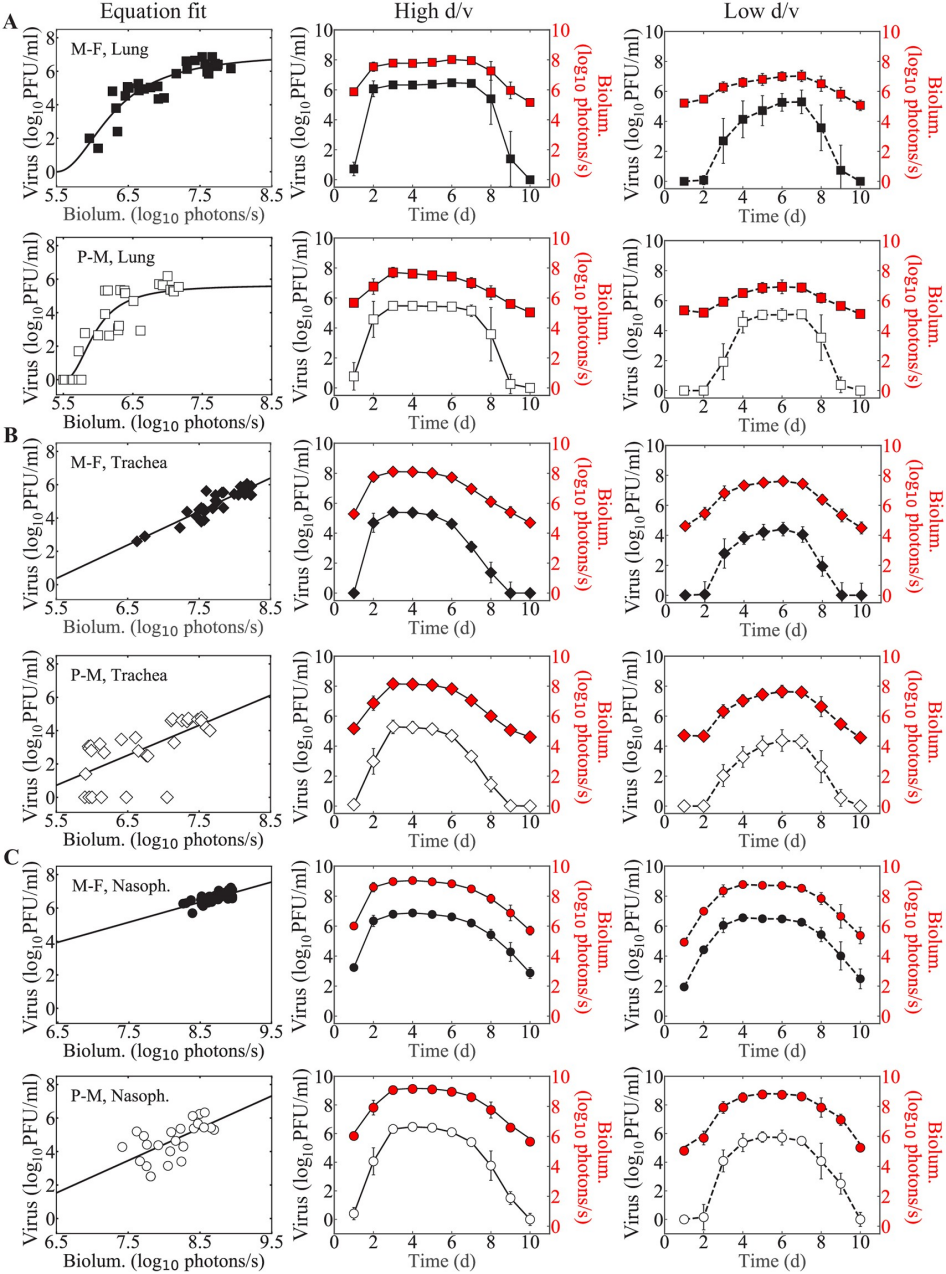
Translation of bioluminescence into viral loads. Fits of Eq (1) or Eq (2) to the paired bioluminescence and viral loads from the lung (Panel A), trachea (Panel B), or nasopharynx (Panel C) of individual mice at 3, 5, or 7 days after infection with low d/v or high d/v of rSeV-luc(M-F*) virus (“M-F”, black) or rSeV-luc(P-M) virus (“P-M”, white). Correlation was derived for each compartment by combining the low d/v and high d/v infections for each strain. Estimated viral load time-series in the lung (Panel A, squares), trachea (Panel B, diamonds), and nasopharynx (Panel C, circles) for infection with rSeV-luc(M-F*) (black) or rSeV-luc(P-M) (white) at high d/v (solid line) or low d/v (dashed line) from the bioluminescence (red). Data are shown as geometric mean ± standard deviation for 5 (paired data) or 15 mice per time point (time series data).

Plotting the bioluminescence data against the corresponding viral loads for individual mice and separately for each virus revealed a nonlinear correlation in the lung data and a linear correlation in the nasopharynx and trachea data (Fig 1). To translate the lung bioluminescence into viral loads, we fit the following Hill-type function to the paired data.

$$\begin{array}{c} V\left(B_{L}\right)=\frac{\alpha B_{L}^{n}}{K_{B_{L}}^{n}+B_{L}^{n}}, \end{array}$$ (1)

where *V* is virus (PFU/ml), *B* is bioluminescence (photons/sec.), *α* is the maximum interaction rate, *K*_*B*_ is the half-saturation constant, and *n* is the Hill coefficient. The subscript ‘L’ denotes the measurements in the lung. We also examined whether a linear function fit the data better and found that Eq (1) gave a better fit based on AIC_c_ (77 versus 86 for the rSeV-luc(M-F*) virus and 95 versus 106 for the rSeV-luc(P-M) virus).

Similarly, to translate the bioluminescence from the trachea and nasopharynx into viral loads, we fit the following linear function to the paired data.

$$\begin{array}{c} V\left(B_{N,T}\right)=\lambda B_{N,T}+\gamma , \end{array}$$ (2)

where λ is the slope (PFU/ml)/(photons/sec.) and *γ* is the intercept (PFU/ml). Subscripts ‘N’ and ‘T’ denote the measurements in the nasopharynx and trachea, respectively. This linear relation is similar to our previous work [14] and gave a better fit compared to nonlinear relation based on AIC_c_ (47 versus 103 for the rSeV-luc(M-F*) virus and 115 versus 119 for the rSeV-luc(P-M) virus in trachea, and 15 versus 19 for the rSeV-luc(M-F*) virus and 74 versus 88 for the rSeV-luc(P-M) virus in nasopharynx).

Each function was fit to the respective paired data using scipy.optimize.curvefit in Python. Fits were performed independently for the rSeV-luc(M-F*) and rSeV-luc(P-M) viruses and estimated viral loads for bioluminescence below the limit of detection (≤ 5.6 log_10_ photons/sec. [16]) were set to 0 log_10_ PFU/ml. Comparison of the bioluminescence and measured or estimated viral loads from the time series and paired data verified the accuracy of the translations (Fig A in S1 Text).

### Viral kinetic model

We used a mathematical model previously developed to describe the biphasic viral load decay during influenza A virus infection [29]. The model tracks 4 populations: susceptible epithelial (“target”) cells (*T*), infected cells in the eclipse phase (*I*_1_), productive infected cells (*I*_2_), and virus (*V*).

$$\frac{dT}{dt}=-\beta TV$$ (3)

$$\frac{dI_{1}}{dt}=\beta TV-kI_{1}$$ (4)

$$\frac{dI_{2}}{dt}=kI_{1}-\delta \left(I_{2}\right)I_{2}$$ (5)

$$\frac{dV}{dt}=pI_{2}-cV$$ (6)

In this model, target cells become infected with virus at rate *β*V per day. Once infected, the cells enter an eclipse phase (I_1_) before transitioning to virus-producing infected cells (I_2_) at a rate k per day. Infected cells produce virus at a rate p PFU/ml/cell/day. Free virus is cleared at a rate c per day. The rate of infected cell clearance changes with their density according to the function *δ*(I_2_) = *δ*_d_/(K_*δ*_ + I_2_), where *δ*_d_/K_*δ*_ is the maximum rate and K_*δ*_ is the half-saturation constant.

### Parameter estimation

Parameters were estimated using a non-linear mixed-effect modeling (NLME) and stochastic approximation expectation minimization (SAEM) algorithm implemented in Monolix 2019R1 [30]. In this approach, each individual parameter is written as $\theta_{i}=\theta e^{\eta_{i}}$, $\eta_{i}=N\left(0,\omega_{i}^{2}\right)$, where *θ* denotes the median value of the parameter in the population and *η*_i_ denotes the random effect that accounts for the inter-individual variability of the parameter within the population. Parameters for each individual were obtained using empirical Bayes estimates, and inter-individual variability was allowed for all parameters with the assumption of an additive error model for the log_10_ viral loads. Estimated viral loads from bioluminescence data below the limit of detection or *V* = 0 log_10_ PFU/ml were left-censored.

Estimated parameters included the rates of virus infection (*β*), virus production (*p*), virus clearance (*c*), eclipse phase transition (*k*), infected cell clearance (*δ*_*d*_), and the half-saturation constant (*K*_*δ*_). The rate of infection (*β*) was allowed to vary between 1 × 10^−9^ − 1.0 (PFU/ml)^−1^d^−1^, the rate of viral clearance between 1 × 10^−2^ − 1 × 10^3^ d^−1^, and the rate of viral production (*p*) between 1 × 10^−2^ − 1 × 10^3^ (PFU/ml) cell^−1^ d^−1^. The eclipse phase transition rate (*k*) bounds were set to 3 d^−1^ and 6 d^−1^ to constrain it within biologically feasible values [29]. The rate of infected cell clearance (*δ*_*d*_) was given a lower limit of 1 × 10^2^ cells d^−1^ and an upper limit of 1 × 10^7^ cells d^−1^. The saturation constant (*K*_*δ*_) was bounded between 1.0 − 1 × 10^7^ cells. The initial number of target cells (*T*_0_) was set to 1 × 10^7^ cells for the lung to maintain consistency with previous studies for high d/v infections in mice [27–29]. The number of susceptible cells in the nasopharynx and trachea was estimated to account for the physiological differences in different parts of the respiratory tract. To do this, the production rate (*p*) was fixed to the value obtained for high d/v infections in the lung. To account for the difference in virus deposition within the respiratory tract due to inoculum volume, we set the value of initial infected cells (*I*_1_(0)) in the nasopharynx, trachea, and lung to 35 cells, 21 cells, and 7 cells for low d/v and to 7 cells, 70 cells, and 700 cells for high d/v, respectively. Similar to previous studies [29], we evaluated other values of *I*_1_(0) and found no significant differences (Table A in S1 Text). The initial number of productively infected cells (*I*_2_(0)) and the initial free virus (*V*_0_) were set to 0. To explore and visualize the regions of parameters consistent with the model, we performed bootstrapping with the model (Eqs (3)–(6)) for each of the infection groups using Rsmlx package [31].

### Statistical analysis

The Welch’s t-test was used to determine the statistical significance of parameter differences among each group of infection with significance established at p<0.05. To compare models, the Akaike Information Criteria with small sample size correction (AIC_c_) was used. The model with the lowest AIC_c_ was considered the best, and ΔAIC_c_ < 2 was considered statistically equivalent [32, 33].

## Results

### Kinetics of bioluminescence and translation into viral loads

In animals infected with either the rSeV-luc(M-F*) or rSeV-luc(P-M) Sendai viruses at a high d/v (7000 PFU in 30 μl), bioluminescence increases in the lung, trachea, and nasopharynx within 2 d of infection (Fig 1). Peak bioluminescence occurs at 2–3 d pi before biphasically decaying and returning to baseline by 10 d pi. Comparatively, in animals infected with a low d/v, bioluminescence was delayed by 2 d with a peak occurring after 4 d pi. Because it is not clear that how much bioluminescence is emitted per infected cell, we translated the bioluminescence into viral loads by fitting Eq (1) to the paired data from the lung and Eq (2) to the paired data from the nasopharynx or trachea independently for the rSeV-luc(M-F*) and rSeV-luc(P-M) viruses (Fig 1 and Table 1). Using the estimated parameters, we then translated the time course bioluminescence data into viral loads for each individual (Fig 1) and verified it against the viral loads from the paired data (Fig A in S1 Text). The translated viral loads in the lung, trachea, and nasopharynx suggested a viral load trend of an initial exponential growth, peak, and biphasic decay. However, the magnitudes and timescales of the viral loads differed depending on virus strain, dose, and respiratory tissue. As expected, the high d/v infections resulted in ∼ 1 log_10_ PFU/ml higher viral peaks with faster growth rates (Fig B and Table B in S1 Text), ∼1 d longer infection duration in the nasopharynx, and ∼2 d earlier peak compared to the low d/v infections in all tissues. High d/v infections in the lung also resulted in faster decay rates (Fig B and Table B in S1 Text), and the rSeV-luc(M-F*) virus showed ∼ 1 log_10_ PFU/ml higher viral peak in the lung and slightly longer infection duration in each tissue compared to the rSeV-luc(P-M) virus.

**Table 1:** Best-fit parameters from translating bioluminescence into viral loads. Parameters obtained by fitting Eq (1) to paired bioluminescence and viral loads from the lung or by fitting Eq (2) to paired bioluminescence and viral loads from the trachea or nasopharynx of individual mice infected with the rSeV-luc(M-F*) virus (“M-F”) or the rSeV-luc(P-M) virus (“P-M”).

Tissue	Virus	Maximum interaction rate, *α* log_10_ (PFU/ml)/(photons/sec.)	Half-saturation constant, *K*_*B*_ log_10_ photons/sec.	Hill coefficient, n
Lung	M-F	2.08	0.80	7.12
	P-M	2.24	0.46	5.66
		Slope, λ log_10_ (PFU/ml)/(photons/sec.)	Intercept, *γ* log_10_ PFU/ml	
Trachea	M-F	2.0	0.37	
	P-M	1.8	0.72	
Nasopharynx	M-F	1.2	2.75	
	P-M	1.9	-0.37	

### Number of infected cells varies with dose and infection site

To quantify the kinetic differences between the two strains, between the high d/v and low d/v infections, and between the lung, trachea, and nasopharyx, we employed a mathematical model (Eqs (3)–(6)) that describes the biphasic decay of viral loads [29]. We first fit the model to the estimated viral loads from the lungs of infected mice while fixing the initial number of target cells (*T*_0_) to the same value for both the high d/v and low d/v infections (i.e., *T*_0_ = 1 × 10^7^cells [27]; Fig C and Table C in S1 Text). Although the model provided a close fit to both data sets and could accurately reproduce both virus- and dose-specific patterns, the resulting parameter estimates for the rate of virus production (*p*) differed between the high d/v and low d/v infections (Table C in S1 Text). Biologically, we anticipated that this parameter could be virus-specific but did not expect it to be dose-dependent. Mathematically, the parameter is dependent on the initial number of target cells where only the product (*pT*_0_) can be reliably estimated [34, 35]. Because the value of *T*_0_ is approximately equivalent to the final number of infected cells [36] and low dose infections likely result in fewer infected cells, we re-fit the model to estimate *T*_0_ for the low d/v infection in the lung while keeping the rate of virus production (*p*) fixed to the value obtained for high d/v infection (i.e., 8.6 PFU/ml/cell/d for the rSeV-luc(M-F*) virus and 0.9 PFU/ml/cell/d for the rSeV-luc(P-M) virus; Table C in S1 Text). The model solutions were indistinguishable visually and statistically (Fig C in S1 Text), and it resulted in a 1 log_10_ lower estimate for the initial number of target cells for the low d/v infections (i.e., *T*_0_ = 1.0 × 10^6^ cells for the rSeV-luc(M-F*) virus and *T*_0_ = 1.1 × 10^6^ cells for the rSeV-luc(P-M) virus; Table D in S1 Text).

We extended this workflow to account for the anatomical differences between the upper and lower respiratory tracts (Table D in S1 Text). The analysis suggested a higher number of target cells in the nasopharynx compared to the trachea (4.5 × 10^6^ cells versus 1.5 × 10^5^ cells for the rSeV-luc(M-F*) virus; p< 1 × 10^−5^, and T_0_ = 1.6 × 10^7^ cells versus *T*_0_ = 1.2 × 10^6^ cells for the rSeV-luc(P-M) virus; p< 1 × 10^−5^) at high d/v infections. For high d/v infections with either virus, *T*_0_ was the highest in the lung (1 × 10^7^ cells for both strains; p< 1 × 10^−5^). For low d/v infections, the estimated *T*_0_ was the lowest in the trachea and highest in the nasopharynx (3.2 × 10^4^ cells and 2.5 × 10^6^ cells for the rSeV-luc(M-F*) virus; p< 1 × 10^−5^, and *T*_0_ = 1.1 × 10^5^ cells and *T*_0_ = 9.0 × 10^6^ cells for the rSeV-luc(P-M) virus; p< 1 × 10^−5^, in the trachea and nasopharynx, respectively).

### Strain-, dose-, and individual-dependent processes

**Fig 2:**
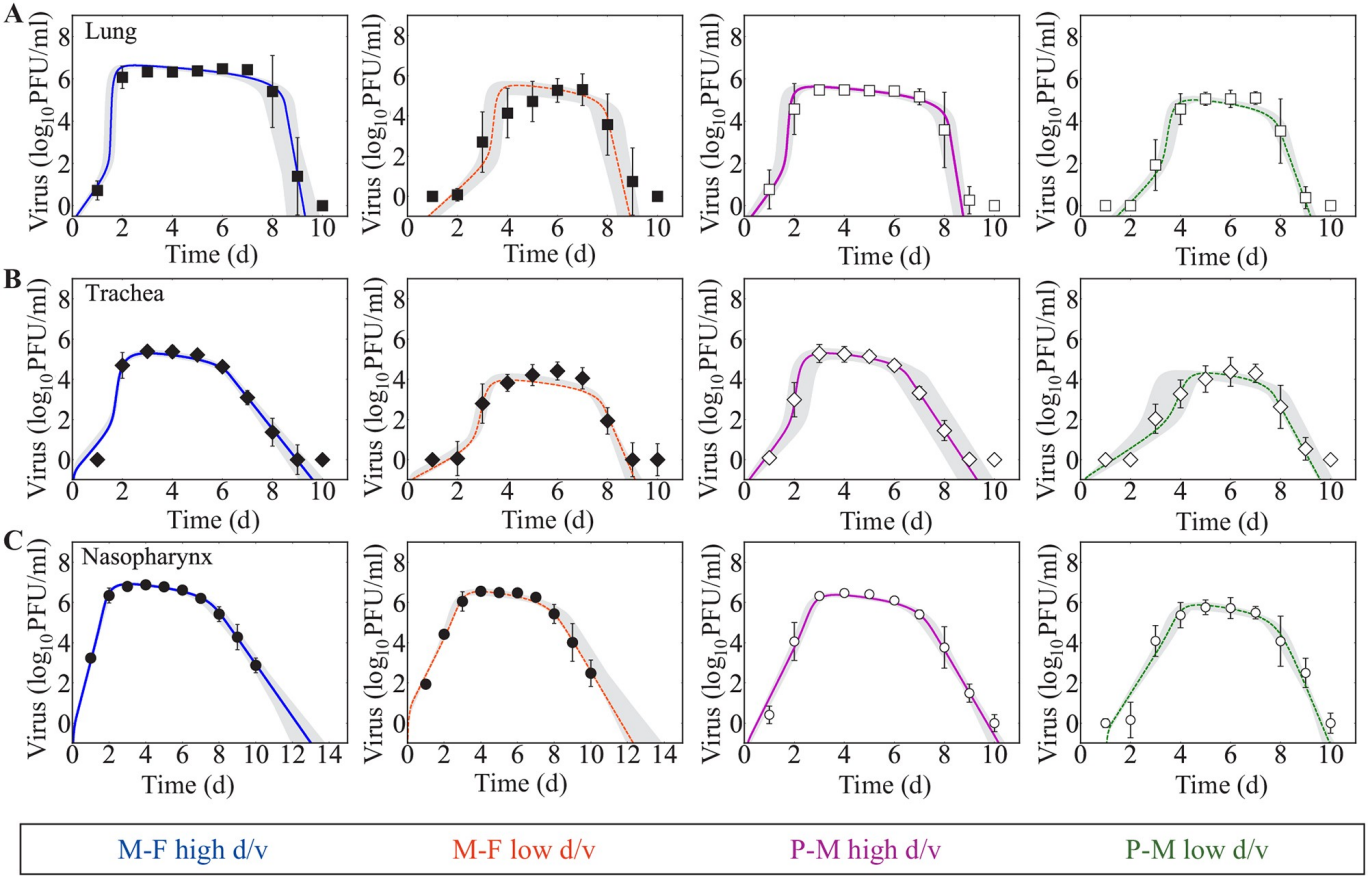
Fit of the viral kinetic model to estimated viral loads from the lung, trachea, and nasopharynx during SeV infection with different strains and doses. (A-C) Fit of the model (Eqs (3)–(6)) to estimated viral loads from the lung (Panel A, squares), trachea (Panel B, diamonds) and nasopharynx (Panel C, circles) of mice infected with rSeV-luc(M-F*) (“M-F”, black) at high d/v (solid blue line) or low d/v (dashed orange line) or with rSeV-luc(P-M) (“P-M”, white) at high d/v (solid magenta line) or low d/v (dashed green line). The gray regions are the model solutions using parameter sets within the 95% CIs. Data are shown as geometric mean ± standard deviation for 15 mice per group.

**Fig 3:**
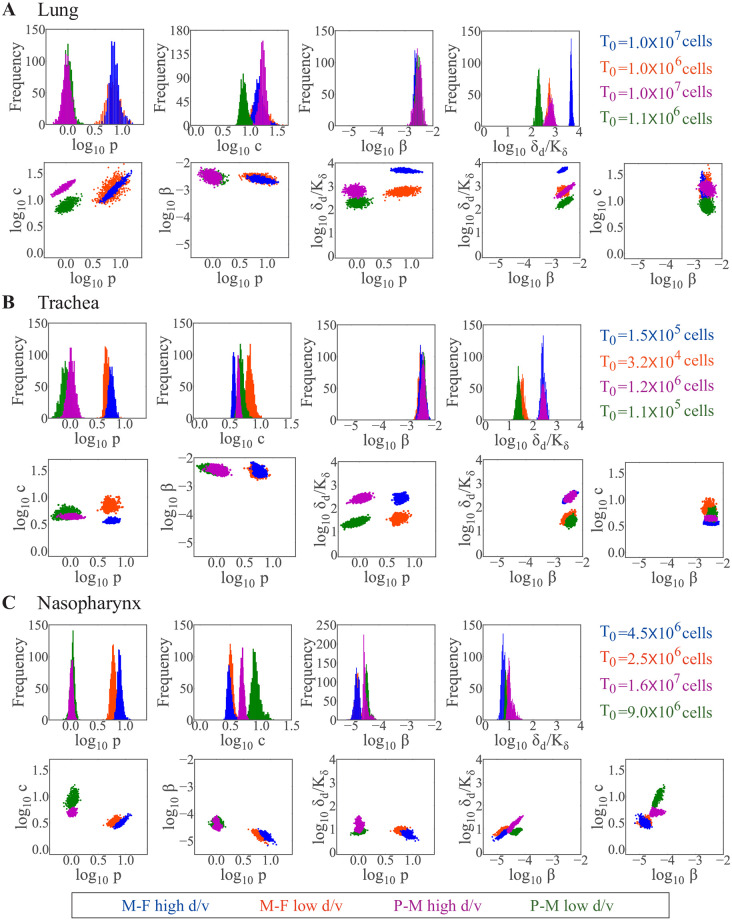
Strain- and dose-dependent parameters in the lung, trachea, and nasopharynx. Comparison of parameter histograms and ensembles resulting from fitting the model (Eqs (3)–(6)) to estimated viral loads from the lung (Panel A), trachea (Panel B) or nasopharynx (Panel C) of mice infected with the rSeV-luc(M-F*) virus (“M-F”) at high d/v (blue) or low d/v (orange), or infected with the rSeV-luc(P-M) virus (“P-M”) at high d/v (magenta) or low d/v (green). Parameters shown are virus production (*p*), virus clearance (*c*), virus infection (*β*), and infected cell clearance (*δ*_*d*_/*K*_*δ*_). Additional histograms and ensemble plots are in Figs E-G in S1 Text.

To effectively identify strain-, dose-, and individual-specific parameters, we fixed the initial number of target cells (*T*_0_) to their estimated values and re-fit the model to each data set (Fig 2 and Table 2 and Fig D in S1 Text). This was sufficient to recover the distribution of the virus production rate (*p*) (Fig 3). Comparing the resulting parameters for each virus showed that the primary strain-specific differences were the rates of virus production (*p*) and infected cell clearance (*δ*_d_/*K*_*δ*_) (Fig 3 and Table 2 and Figs E-G in S1 Text). As expected, the rSeV-luc(P-M) virus, which exhibits an attenuated phenotype, had a lower virus production rate (∼0.9 PFU/cell/d) compared to the rSeV-luc(M-F*) virus (∼8.6 PFU/cell/d). In the lung (Fig 3A), the maximum rate of infected cell clearance (*δ*_*d*_/*K*_*δ*_) for the rSeV-luc(M-F*) virus was increased in high d/v infections (4.6 × 10^3^ d^−1^) compared to low d/v infections (5.4 × 10^2^ d^−1^; p<1 × 10^−5^) and compared to the rSeV-luc(P-M) virus at high d/v (6.2 × 10^2^ d^−1^; p<1 × 10^−5^). At low d/v, this rate was lowest for the rSeV-luc(P-M) virus (1.6 × 10^2^ d^−1^) compared to the high d/v and to the rSeV-luc(M-F*) virus (p<1 × 10^−3^ for both). The majority of the other parameters (i.e., virus infection (*β*), eclipse phase (*k*), and virus clearance (*c*)) were similar for all the infection groups in the lung with the exception of the rate of virus clearance. This parameter was significantly lower for the rSeV-luc(P-M) virus at low d/v than at high d/v (7.4 d^−1^ versus 16 d^−1^; p<1 × 10^−3^). In the trachea (Fig 3B), there were small differences in the maximum rate of infected cell clearance between the two strains but statistical significance was not reached (high d/v, p = 0.09; low d/v, p = 0.07). In contrast, there was a dose dependency in this parameter, where it was larger in the high d/v infections (p<1 × 10^−3^ for both strains). In the nasopharynx (Fig 3C), the infected cell clearance rate was neither strain- nor dose-dependent. However, the rates of virus infection (*β*) and clearance (*c*) were different between the two strains at both doses (p<0.01 for both parameters).

**Table 2:** Maximum likelihood estimates of population parameters. Population parameters (median values) and 95% confidence intervals obtained from fitting the model (Eqs (3)–(6)) to estimated viral loads from the lung, trachea, or nasopharynx of mice infected with either the rSeV-luc(M-F*) virus (“M-F”) or the rSeV-luc(P-M) virus (“P-M”) at a high d/v or low d/v. The initial numbers of target cells (*T*(0)) and infected cells (*I*_1_(0)) were fixed to the indicated value, and the initial number of productively infected cells (*I*_2_(0)) and the initial virus (*V*(0)) were set to 0.

	Virus	Dose PFU	Virus production, *p* (PFU/ml) cell^−1^ d^−1^	Virus clearance, *c* d^−1^	Virus infection, *β* (PFU/ml)^−1^ d^−1^ × 10^−3^	Eclipse phase, *k* d^−1^	Infected cell clearance, *δ*_*d*_ cell^−1^d^−1^ × 10^5^	Half-saturation constant, *K*_*δ*_ cells × 10^4^	Initial infected cells, *I*_1_(0) cells	Initial target cells, *T*(0) cells	Basic reproduction number, *R*_0_
**Lung**	**M-F**	**70**	8.2	17	2.5	3.0	1.9	0.035	7	1.0×10^6^	2.2
			[4.0–12]	[9.0–28]	[1.8–3.8]	[2.9–3.2]	[1.8–2.3]	[0.023–0.052]	-	-	[1.9–2.5]
		**7000**	8.6	17	2.1	3.5	14	0.03	700	1.0×10^7^	2.3
			[5.0–12]	[10–24]	[1.8–3.0]	[3.1–4.8]	[13–14]	[0.025–0.035]	-	-	[2.1–2.9]
	**P-M**	**70**	0.8	7.4	3.5	3.0	2.4	0.14	7	1.1×10^6^	2.4
			[0.7–1.4]	[6.0–10]	[1.9–4.4]	[2.9–3.0]	[2.4–3.0]	[0.092–0.21]	-	-	[2.0–2.4]
		**7000**	0.9	16	3.1	3.0	15	0.23	700	1.0×10^7^	2.6
			[0.6–1.3]	[12–21]	[1.7–5.0]	[3.0–3.1]	[14–15]	[0.14–0.41]	-	-	[2.4–3.0]
**Trachea**	**M-F**	**70**	8.0	5.4	2.9	3.0	0.06	0.013	21	3.2×10^4^	2.9
			[7.0–9.0]	[5.4–8.8]	[2.1–4.7]	[2.9–3.0]	[0.057–0.067]	[0.01–0.025]	-	-	[1.8–2.2]
		**7000**	7.9	3.6	3.4	3.0	0.24	0.01	70	1.5×10^5^	4.6
			[6.7–8.5]	[3.4–4.0]	[2.9–4.0]	[2.9–3.0]	[0.23–0.25]	[0.01–0.02]	-	-	[3.8–4.3]
	**P-M**	**70**	1.0	4.9	4.1	3.0	0.20	0.05	21	1.1×10^5^	2.3
			[0.5–1.2]	[4.0–6.2]	[2.8–5.1]	[2.9–3.0]	[0.16–0.20]	[0.05–0.11]	-	-	[2.0–2.4]
		**7000**	1.0	4.3	4.1	3.0	2.5	0.084	70	1.2×10^6^	3.8
			[0.7–1.4]	[3.9–4.7]	[2.6–5.3]	[2.9–3.0]	[2.2–3.0]	[0.066–0.14]	-	-	[3.5–4.7]
**Nasopharynx**	**M-F**	**70**	7.4	3.5	0.011	3.0	4.8	7.5	35	2.5×10^6^	9.0
			[5.0–7.8]	[2.7–3.8]	[0.01–0.02]	[2.9–3.0]	[3.8–4.9]	[3.5–7.2]	-	-	[7.2–9.8]
		**7000**	7.6	3.0	0.019	3.0	7.7	10	7	4.5×10^6^	28.1
			[6.6–10]	[2.6–3.8]	[0.011–0.022]	[2.9–3.1]	[7.5–8.7]	[9.9–20]	-	-	[27–37]
	**P-M**	**70**	1.0	7.7	0.039	3.0	22	30	35	9.0×10^6^	6.2
			[0.8–1.1]	[6.5–8.2]	[0.031–0.045]	[2.9–3.0]	[13–27]	[15–37]	-	-	[5.2–7.2]
		**7000**	1.0	4.9	0.028	3.0	35	34	7	1.6×10^7^	8.8
			[0.9–1.1]	[4.3–5.6]	[0.025–0.061]	[2.9–3.0]	[31–38]	[13–47]	-	-	[7.7–12.2]

To assess the parameters driving inter-individual variability, we examined the estimated standard deviation (*ω*) of the random effect for each parameter and the individual fits (Table E and Figs L-W in S1 Text). The largest deviance that accounted for the variability in the SeV dynamics within the lung was in the infected cell clearance parameters (*δ*_*d*_ and *K*_*δ*_). There was little variability in parameters for infection in the trachea and nasopharynx.

Similar to our previous work for influenza [29], two main parameter correlations were evident. These were between the rates of virus production (*p*) and clearance (*c*) and between the rates of infection (*β*) and infected cell clearance (*δ*_*d*_/*K*_*δ*_). Despite the aforementioned differences, the basic reproduction number (R_0_ = *β*pK_*δ*_T_0_/*c*δ_d_ [29]) was similar within the lung for each strain and dose (R_0_ 2.0 − 2.7; Table 2). R_0_ was maximal in the high d/v scenarios within the other respiratory tissues (3.8 − 4.6 versus 2.3 − 2.9 in the trachea for both viruses, p<1 × 10^−3^; 28 versus 9 (rSeV-luc(M-F*)) and 8.8 versus 6.2 (rSeV-luc(P-M)) in the nasopharynx, p<1 × 10^−3^; Table 2).

### Respiratory tissue-dependent processes

**Fig 4:**
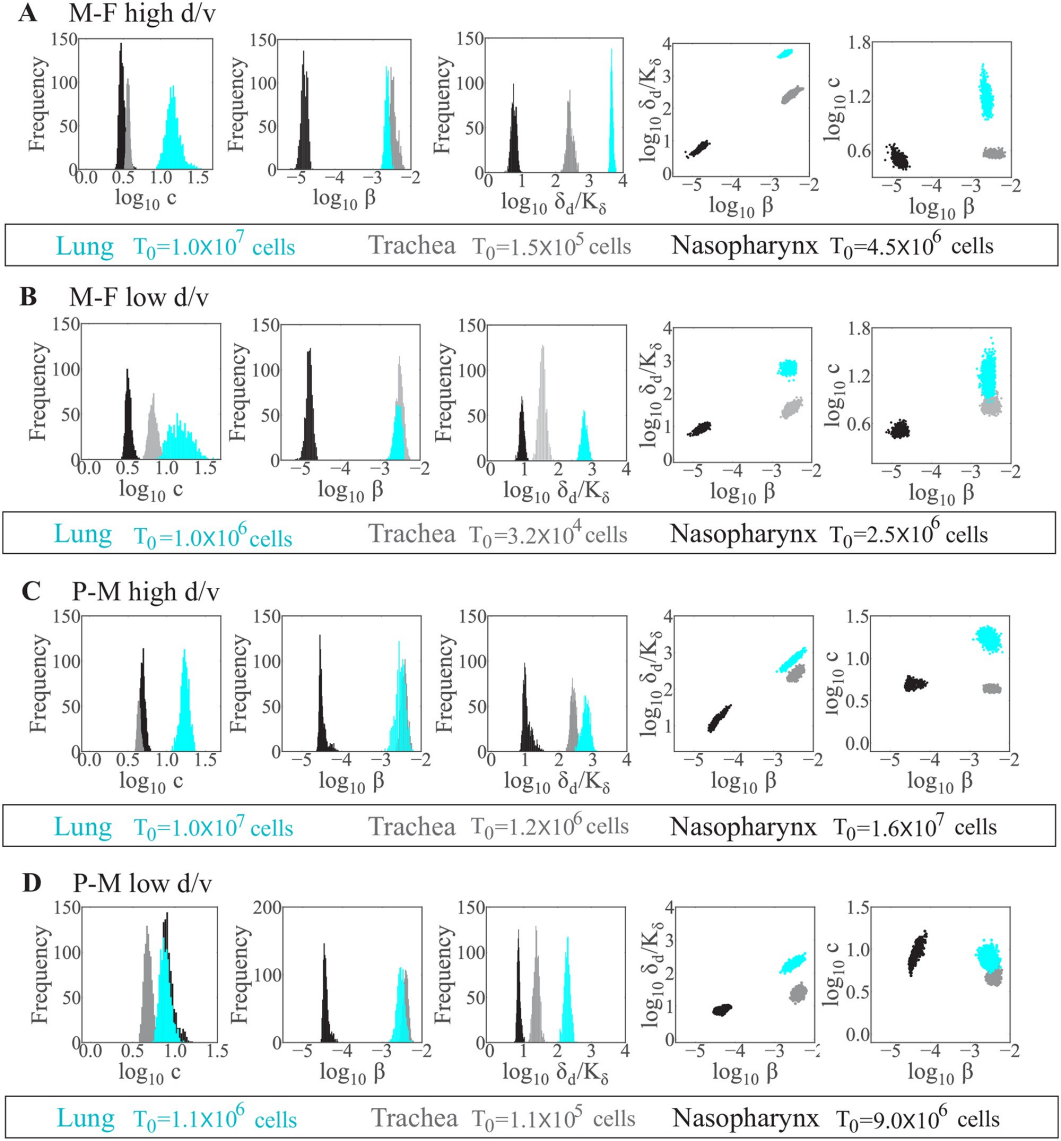
Respiratory tissue-dependent parameters. Comparison of parameter histograms and ensembles resulting from fitting the model (Eqs (3)–(6)) to estimated viral loads from the lung (cyan), trachea (gray), or nasopharynx (black) of mice infected with the rSeV-luc(M-F*) virus (“M-F”) at high d/v (Panel A) or low d/v (Panel B), or infected with the rSeV-luc(P-M) virus (“P-M”) at high d/v (Panel C) or low d/v (Panel D). Parameters shown are virus clearance (*c*), virus infection (*β*), and infected cell clearance (*δ*_*d*_/*K*_*δ*_). Additional histograms and ensemble plots are in Figs H-K in S1 Text.

Comparing the resulting parameters between the lung, trachea, and nasopharynx, the primary tissue-dependent alteration was in the maximum rate of infected cell clearance (*δ*_*d*_/*K*_*δ*_) (Fig 4 and Figs H-K in S1 Text). This parameter was significantly higher in the lung (4.6 × 10^3^ d^−1^) than in the trachea (2.4 × 10^2^ d^−1^; p< 1 × 10^−5^) and nasopharynx (7.7 d^−1^; p< 1 × 10^−5^) for the rSeV-luc(M-F*) virus at high d/v (Fig 4A). The same trend was also observed for the remaining infection groups for this parameter (Fig 4B–4D). The rate of virus infection (*β*) was similar between the lung and trachea but significantly lower in the nasopharynx (p< 1 × 10^−5^ for both). The rate of virus clearance (*c*) was the highest in the lung for all infection groups with the exception of rSeV-luc(P-M) virus infection at low d/v (p<1 × 10^−5^ for all groups). The basic reproduction number (R_0_) was significantly higher in the nasopharynx than in the lung or trachea for all infection groups (p< 1 × 10^−5^ for all groups; Table 2).

## Discussion

**Fig 5:**
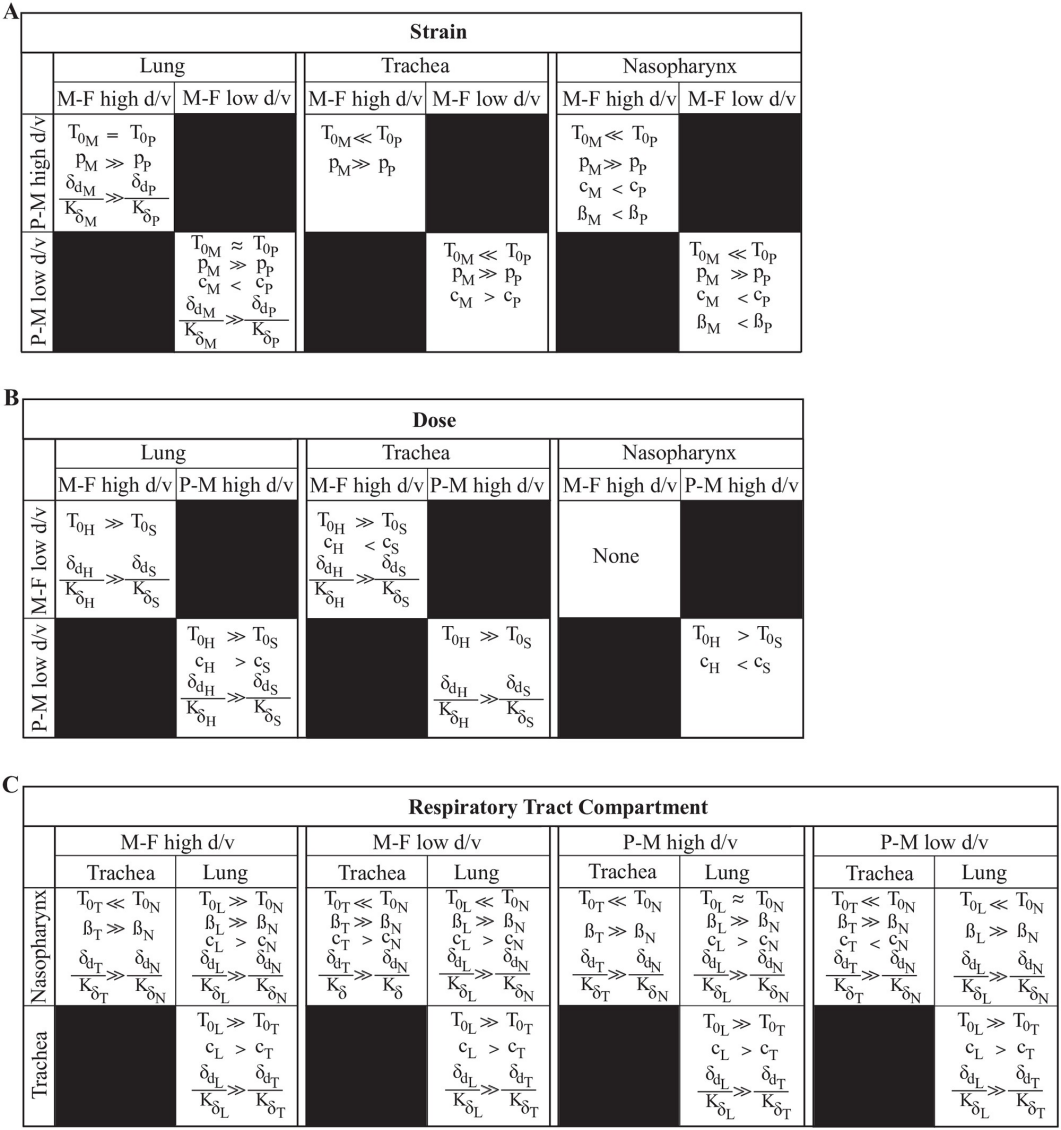
Summary of strain-, dose- and tissue-dependent processes. Summary of parameters that were distinct between different infection scenarios. Parameters consistently different included (A) strain-dependent virus production rates (*p*), (B-C) dose- and tissue-dependent infected cell clearance rates (*δ*_*d*_/*K*_*δ*_). Other parameters shown include the initial number of target cells (*T*_0_), virus clearance (*c*), and virus infection (*β*). Subscripts “M” and “P” denote the rSeV-luc(M-F*) (“M-F”) and rSeV-luc(P-M) (“P-M”) viruses, respectively. Subscripts “L”, “T”, and “N” denote the lung, trachea, and nasopharynx, respectively. Subscripts “H” and “S” denote high d/v and low d/v, respectively.

Parainfluenza virus infections can target different areas of the respiratory tract and tend to increase in severity with greater impact on the lung, which can lead to bronchiolitis, pneumonia, and hospitalization [5, 37]. However, most infections are mild with the virus primarily infecting the upper respiratory tract without progressing to the lower airways [38, 39]. Many factors likely contribute to this heterogeneity, including serotype, strain, dose, immune status, and age. Our analysis of different infection scenarios suggested that only a few selected processes drive distinct HPIV dynamics due to strain, doses, individual, and/or site of infection (upper versus lower airways; summarized in Fig 5).

Our analyses were able to verify that the rSeV-luc(P-M) virus, which produces an attenuated phenotype [14], replicates more slowly (lower *p*) than the wild-type-like strain, rSeV-luc(M-F*) (Fig 3). In the lung, this was paired with slower infected cell clearance (Fig 5A). The change in infected cell clearance was evident in several of the infection scenarios and a major driver of the dose- and tissue-specific differences (Fig 5B and 5C). This rate was also the most variable amongst individuals, which was unsurprising given that the model dynamics are highly sensitive to changes in this parameter [29]. Our previous studies on influenza virus infection found that this rate predominantly reflects the expansion rate of CD8^+^ T cells [40]. Thus, a slower infected cell clearance rate would indicate that fewer CD8^+^ T cells are recruited to the infected area. Reduced B cells and antibody generation may accompany these changes. This is consistent with dose-dependent experimental studies on other viruses [41, 42] and our own study on SeV infection [16] where fewer pulmonary T cells and B cells were observed in low d/v infections compared to high d/v infections. Although the relative distribution and functionality of CD8^+^ T cells in different areas of the respiratory tract is mostly unknown [43], some evidence suggests differing phenotypes are present in the trachea and lung [44] and that tissue tropism drives their patterns [45]. Interestingly, our model suggests that the rates of infected cell clearance and viral infectivity (*β*) are correlated [29], and that both of these rates are highest in the lung and lowest in the nasopharynx (Figs 4 and 5). This same trend was evident in the rate of viral clearance (*c*), which could be related to spatially-variable antibody concentrations within the respiratory tissue [46, 47].

Depending on the timing of the CD8^+^ T cell and neutralizing antibody responses or in immunocompromised hosts, it is possible that the correlation between bioluminescence and viral loads could deviate. The data used here were obtained from an acute infection in immunocompetent, naive mice [16], and bioluminescence in this system reflects the number of infected cells rather than extracellular virus because it is dependent on reporter gene expression and measured only when the viral genome is translated [14]. We chose to estimate the viral loads because it is not known how bioluminescence and, consequently, virus production in a single cell changes over its infected lifetime. That is, increases in bioluminescence may not directly translate to an increase in the number of infected cells. This is likely why the relation between these two entities in the lung, which is denser than the trachea or nasopharynx, was nonlinear. Our previous work on influenza virus infection in the lung showed that our model can accurately estimate the infected cell kinetics throughout the infection by using viral load data [40]. However, further studies would be needed to determine how the relation between bioluminescence and viral loads might change with alter immunologic environments.

Fitting the model to viral load data from different tissues in the upper and lower respiratory tracts required altering the number of target cells (*T*_0_) to recover the same virus production rate (*p*). While it is possible that the rate could vary across tissues or even within a tissue, the estimated number of target cells in the URT and LRT (Table D in S1 Text) were consistent with the relative differences in surface area of the murine respiratory tract [48–50]. Further, the variation in dosing volume was also captured where the low volume resulted in a higher number of infected cells in the nasopharynx compared to the lung while the high volume resulted in a larger number of infected cells in the lung compared to the low volume. Although it is readily apparent that less virus (i.e., from lower doses) would infect fewer cells, the differences in *T*_0_ between each virus in some tissues (e.g., 1.5 × 10^5^ cells (rSeV-luc(M-F*)) versus 1.2 × 10^6^ cells (rSeV-luc(P-M)) in the trachea) could be attributed to other mechanisms. Host responses, such as type I IFN, play a role in limiting virus spread [51–53] and HPIV serotypes have been reported to differ in their ability to induce cytokine production [7, 54]. Including innate immune responses within a model can limit the number of infected cells [55] and some studies have suggested that these may help to investigate dose-dependent kinetics for some viruses [56, 57]. However, the effects seem to be relatively small and additional studies would be necessary to investigate the contribution from specific immune components.

A low volume (5μl) was used to mimic the progression of an URT infection to the LRT and would suggest that the majority of the inoculum is deposited in the nasopharynx with little reaching the lower airways. Indeed, the low d/v infection was initially restricted to the upper respiratory tract and only visible in the lung after ∼2 days while virus was immediately apparent in both the upper and lower respiratory airways during high d/v infections (Fig 1) [16]. Here, we modeled the nasopharynx, trachea, and lung independently and assumed viral transport within the respiratory tract played a minimal role in the viral kinetics. Modeling studies that have taken this into account found that the rates of virus transport to and from each tissue are relatively negligible or unidentifiable [58–61]. Although spatial structure, even within a tissue, can yield spatially-dependent dynamics (e.g., as in [58]), we were still able to recover reasonable estimates of the relative infection sizes by ensuring similar virus replication rates (*p*) throughout the entire respiratory tract. That is, the high d/v infections yielded only ∼1.8x more infected cells in nasopharynx where we would anticipate similar or slightly higher infection levels as in low d/v infections (Table 2). Comparatively, our model estimated that ∼10x more cells were infected in the lung. In addition, the values of *T*_0_ reflected the differences in surface area of the respiratory tract where the lung and nasopharynx are significantly larger than the trachea [48–50].

Although the low dose resulted in slightly fewer infected cells in the nasopharynx, there was little change in the other processes compared to the high dose (Fig 3C) and little individual heterogeneity within this tissue (Table E in S1 Text). This is likely because it is the first contact site of the virus and may not be a particularly sensitive location to detect these types of changes. This may help explain the lack of heterogeneity observed in clinical symptoms with respect to the HPIV serotype [62]. These findings also support the idea that sampling of nasal or throat tissue may not reflect disease, which is typically more linked to the impact on the lower respiratory tract [40, 63–66].

In the data used here, there was minimal weight loss in the animals infected with low d/v compared to high d/v [16], which indicates reduced disease severity. We previously established that disease severity (as measured by weight loss) is nonlinearly connected with pathological findings, including the extent of virus-mediated lung damage and inflammation [40]. In addition, we discovered that each of these metrics could be approximated using the infected cell dynamics (e.g., cumulative area under the curve of *I*_2_ approximates the lung damage). Thus, our finding that the number of infected cells was significantly lower in the low d/v groups is in accordance with the reduced weight loss in these animals. In general, having fewer infected cells and/or reduced activation of host responses should lessen virus-induced lung damage and immunopathology.

Distinguishing the drivers of HPIV infection heterogeneity and the impact of different strains, doses, patients, and sites of infection as we did here is central to understanding the disease course and developing effective treatments. It may also help identify the mechanisms that influence disproportionate disease in children and the immunocompromised, who may develop more severe presentations from otherwise low doses. In addition, although transmission dynamics are complex and typically associated with viral presence in the URT, further insight into tissue-specific viral dynamics and the possibility of prolonged virus shedding is vital to abrogate the disease [16, 67, 68].

## Supporting information

S1 Text Supplementary text. Supplemental text, figures, and tables.
(PDF)
