# Supplementary material for: Quantifying dose-, strain-, and tissue-specific kinetics of parainfluenza virus infection
Source: PLoS Comput Biol. 2021 Aug 12;17(8):e1009299. doi: 10.1371/journal.pcbi.1009299 (PMC8384156; doi:10.1371/journal.pcbi.1009299)
Supplement: S1 Text — Supplemental text, figures, and tables. (PDF) [file pcbi.1009299.s001.pdf]

Supplementary Material

Quantifying Dose-, Strain-, and Tissue-Specific Kinetics of Parainfluenza Virus Infection

Lubna Pinky[1], Crystal W. Burke[2], Charles J. Russell[3], and Amber M. Smith[1,*]

[1]Department of Pediatrics, University of Tennessee Health Science Center, Memphis, Tennessee, USA

[2] United States Army Medical Research Institute for Infectious Diseases, Fort Detrick, Maryland, USA

[3] Department of Infectious Diseases, St. Jude Children's Research Hospital, Memphis, Tennessee, USA

[*]Corresponding Author: amber.smith@uthsc.edu

Verifying Translation of Bioluminescence to Viral Load

To verify the accuracy of the translation of bioluminescence to viral loads, we plotted bioluminescence and estimated viral loads from the time series data against the bioluminescence and viral loads from the paired data (Fig A).

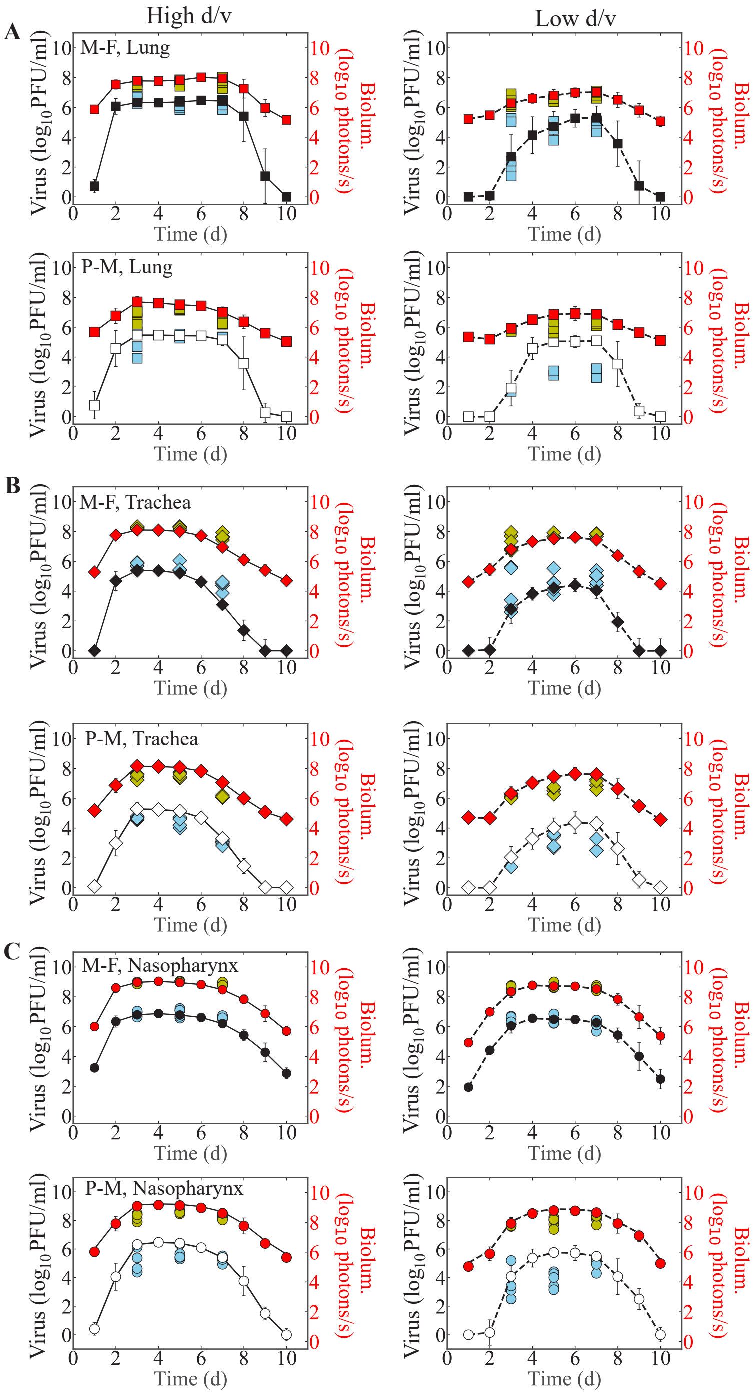

Figure A: Overlay of bioluminescence and corresponding measured or estimated viral loads. Bioluminescence from the paired data (green) and time series data (red) together with the measured viral loads (paired; cyan) and estimated viral loads (time series; black/white) from the lung (Panel A; squares), trachea (Panel B; diamonds) or nasopharynx (Panel C, circles) for infection with rSeV-luc(M-F*) (black) or rSeV-luc(P-M) (white) at high d/v (solid line) or low d/v (dashed line).

Impact of Varying the Initial Number of Infected Cells

To evaluate the impact of the initial number of infected cells ($I_1(0)$) on parameter estimates, we fit the model (Eqs (3)-(6), Main Text) to the estimated viral loads from the lungs, trachea or nasopharynx of mice infected with either a low d/v or high d/v of the rSeV-luc(M-F*) virus or the rSeV-luc(P-M) virus using different values of initial number of infected cells indicated in Table A. These results show that there were no significant differences in the model parameters.

Table A: Parameter estimates when varying the initial number of infected cells, $I_1(0)$. Estimated population parameters obtained from fitting the model (Eqs (3)-(6), Main Text) to the estimated viral loads from the lungs of mice infected with either a low d/v or high d/v of the rSeV-luc(M-F*) virus ("M-F") or the rSeV-luc(P-M) virus ("P-M").

	**Virus**	**Dose** PFU	**Initial infected cells** $I_1(0)$, cells	**Virus production,** p (PFU/ml) cell^{-1} d^{-1}	**Virus clearance,** c d^{-1}	**Virus infection,** β (PFU/ml)$^{-1}$ d^{-1} $\times 10^{-3}$	**Eclipse phase,** k d^{-1}	**Infected cell clearance,** δ_d cell^{-1}d^{-1} $\times 10^{5}$	**Half-saturation constant,** K_δ cell $\times 10^{3}$
**Lung**	**M-F**	**70**	7	8.2	17.0	2.5	3.0	1.9	0.035
			20	7.5	20.0	2.8	3.0	1.9	0.035
			40	7.8	19.6	2.6	3.0	1.9	0.033
		**7000**	700	8.6	17.0	2.1	3.5	14	0.030
			2000	7.4	14.5	2.3	3.0	14	0.029
			4000	7.4	15.5	2.2	3.0	14	0.028
	**P-M**	**70**	7	0.80	7.4	3.5	3.0	2.4	0.14
			20	0.78	7.5	3.5	3.0	2.4	0.13
			40	0.74	7.4	3.4	3.0	2.4	0.13
		**7000**	700	0.9	16.0	3.1	3.0	15	0.23
			2000	1.1	18.8	2.9	3.0	15	0.19
			4000	0.9	18.0	3.1	3.0	14	0.19
**Trachea**	**M-F**	**70**	7	8.2	5.9	3.2	3.0	0.06	0.015
			21	8.0	5.4	2.9	3.0	0.06	0.013
			70	7.2	6.2	3.0	3.0	0.06	0.012
		**7000**	7	8.2	3.5	4.0	3.0	0.25	0.010
			70	7.9	3.6	3.4	3.0	0.24	0.010
			200	7.0	3.7	2.9	3.0	0.23	0.011
	**P-M**	**70**	7	1.0	5.0	4.9	3.0	0.20	0.05
			21	1.0	4.9	4.1	3.0	0.20	0.05
			70	0.78	5.0	4.9	3.0	0.20	0.05
		**7000**	7	1.0	4.3	4.5	3.0	2.5	0.112
			70	1.0	4.3	4.1	3.0	2.5	0.084
			200	0.9	4.3	4.4	3.0	2.4	0.076
**Nasopharynx**	**M-F**	**70**	7	7.9	3.7	0.014	3.0	4.6	7.4
			35	7.4	3.5	0.011	3.0	4.8	7.5
			70	8.0	3.3	0.010	3.0	4.4	6.4
		**7000**	7	7.6	3.0	0.019	3.0	7.7	10
			70	7.3	2.9	0.016	3.0	7.5	12
			200	7.2	2.9	0.021	3.0	7.2	20
	**P-M**	**70**	7	0.94	7.1	0.055	3.0	21	27
			35	1.0	7.7	0.039	3.0	22	30
			70	0.9	7.0	0.039	3.0	22	28
		**7000**	7	1.0	4.9	0.028	3.0	35	34
			70	0.9	4.8	0.055	3.0	32	40
			200	0.9	4.8	0.058	3.0	32	44

Linear Regression Analysis

To quantify the rates of virus growth and decay in each phase of the viral load data, we performed a linear regression analysis using the function scipy.optimize.curvefit in Python (Table B, Fig B). The results show that high d/v infections resulted in higher rates of virus growth compared to the low d/v infections (e.g., 5.3 versus 2.0 $\log_{10}$ PFU/ml d^{-1} for the rSeV-luc(M-F*) virus in the lung; p=0.008). In addition, the rate of virus decay in the second phase was significantly faster in the lung for infection with the rSeV-luc(M-F*) virus at high d/v. There were no significant differences in the rates of virus growth or decay in the nasopharynx for either viruses or doses ($p > 0.05$).

Table B: Best-fit parameters from the linear regression analysis. Parameters from the linear regression analysis of the estimated viral loads data from lung, trachea and naspharynx of mice infected with rSeV-luc(M-F*) virus ("M-F") or with rSeV-luc(P-M) virus ("P-M") at high d/v or low d/v during the virus growth phase (1-2 d pi for high d/v and 2-4 d pi for low d/v), the first virus decay phase (2-7 d pi for high d/v and 4-7 d pi for low d/v), and the second decay phase (7-9 d pi).

	**Virus**	**Dose** PFU	**Growth phase** $\log_{10}$ (PFU/ml) d^{-1}	**First phase of decay** $\log_{10}$ (PFU/ml) d^{-1}	**Second phase of decay** $\log_{10}$ (PFU/ml) d^{-1}
**Lung**	**M-F**	70	2.0	0.55	-2.2
		7000	5.3	0.03	-4.0
	**P-M**	70	2.2	0.02	-2.3
		7000	3.8	-0.07	-3.3
**Trachea**	**M-F**	70	1.8	0.20	-1.27
		7000	4.7	-0.09	-1.22
	**P-M**	70	1.6	0.35	-1.80
		7000	2.8	-0.07	-1.84
**Nasal**	**M-F**	70	2.0	-0.04	-1.27
		7000	3.1	-0.06	-1.22
	**P-M**	70	2.6	-0.03	-1.80
		7000	3.6	-0.07	-1.84

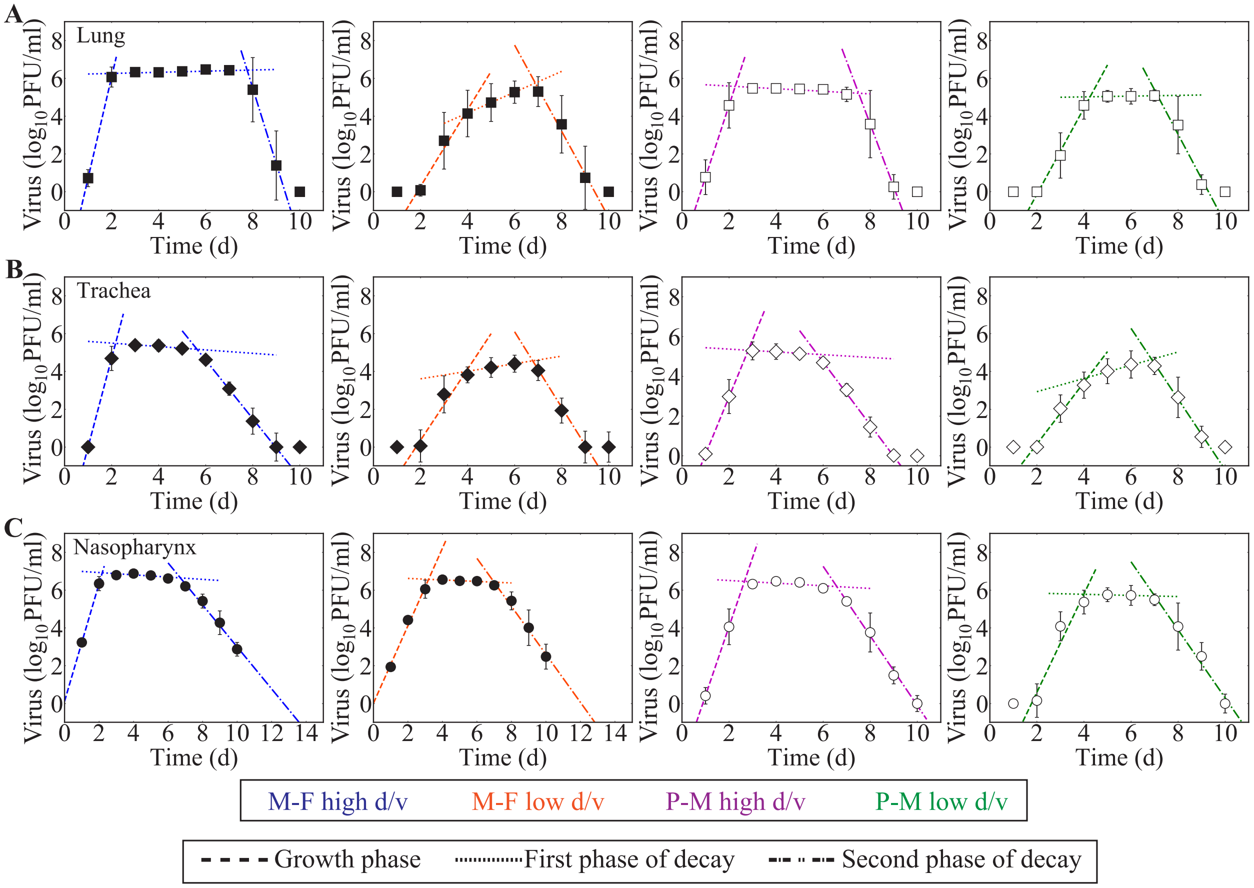

Figure B: Linear regression analysis. Fits obtained from the linear regression analysis of the viral growth (1-2 d pi for high d/v and 2-4 d pi for low d/v; dashed line), first viral decay phase (2-7 d pi for high d/v and 4-7 d pi for low d/v; dotted line), and second viral decay phase (7-9 d pi; dash dotted line) from the lungs (Panel A; squares), trachea (Panel B; diamonds), and nasopharynx (Panel C; circles) of mice infected with rSeV-luc(M-F*) (black) at high d/v (blue lines) or low d/v (orange lines) or with rSeV-luc(P-M) (white) at high d/v (magenta lines) or low d/v (green lines). Parameters are in Table B.

Model Fits With Equivalent Numbers of Target Cells

To assess the differences in model parameters when keeping the initial number of target cells (i.e., $T_0 = 1 \times 10^7$ cells) equivalent between the high d/v and low d/v, we fit the model (Eqs (3)–(6), Main Text) to the estimated lung viral loads. This resulted in a lower rate of virus production (p) in low d/v infection compared to high d/v infection (Table C).

Table C: Population parameter estimates (lung) using equivalent values of T_0. Estimated population parameters and 95% confidence intervals obtained from fitting the model (Eqs (3)-(6), Main Text) to the estimated viral loads from the lungs of mice infected with either a low d/v or high d/v of the rSeV-luc(M-F*) virus ("M-F") or the rSeV-luc(P-M) virus ("P-M") while fixing the initial number of target cells to $T_0 = 1 \times 10^7$ cells.

	Virus	Dose PFU	Virus production, p (PFU/ml) cell^{-1} d^{-1}	Virus clearance, c d^{-1}	Virus infection, β (PFU/ml)$^{-1}$ d^{-1} $\times 10^{-3}$	Eclipse phase, k d^{-1}	Infected cell clearance, δ_d cell^{-1}d^{-1} $\times 10^6$	Half-saturation constant, K_δ cell $\times 10^3$
**Lung**	**M-F**	**70**	0.2 [0.1–0.5]	12 [8.6–16]	2.2 [1.8–5.5]	3.0 [2.9–3.0]	2.0 [1.8–2.3]	10 [4.2–12]
		**7000**	8.6 [5.0–12]	17 [10–24]	2.1 [1.8–3.0]	3.5 [3.1–4.8]	1.4 [1.36–1.48]	0.30 [0.25–0.35]
	**P-M**	**70**	0.1 [0.1–0.2]	7.9 [6.3–10]	2.9 [1.7–4.5]	3.0 [2.9–3.0]	2.1 [1.9–2.3]	12 [8.0–20]
		**7000**	0.9 [0.6–1.3]	16 [12–21]	3.1 [1.7–5.0]	3.0 [3.0–3.1]	1.5 [1.43–1.58]	2.3 [1.4–4.1]

Estimating the Initial Number of Target Cells

To estimate the initial number of target cells (T_0) in low d/v infections that would result in a similar rate of virus production (p) as the high d/v, we fixed the rate of virus production to the value obtained from fitting the model to the high d/v data (i.e., 8.6 (PFU/ml) d^{-1} for rSeV-luc(M-F*) and 0.9 (PFU/ml) d^{-1} for rSeV-luc(P-M)) and re-fit the model. The resulting model fits were indistinguishable visually (Fig C) and statistically based on the Akaike Information Criteria with small sample size correction (AIC$_c$; 365 (fixed T_0) and 364 (estimated T_0) for the rSeV-luc(M-F*) virus and 298.2 (fixed T_0) and 297.3 (estimated T_0) for the rSeV-luc(P-M)) virus). This analyses suggested that low d/v infections have fewer target cells (Table D).

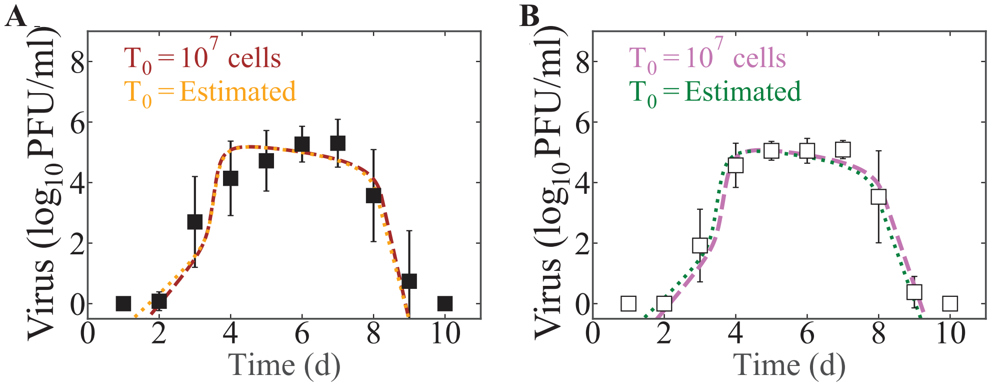

Figure C: Comparison of model fits from estimating and fixing the initial number of target cells (T_0) for low d/v infections in the lung. Fit of the model (Eqs (3)-(6), Main Text) to the estimated viral loads from the lungs of mice infected with low d/v of the rSeV-luc(M-F*) ("M-F") virus (Panel A) or rSeV-luc(P-M) ("P-M") virus (Panel B). Fits were done with the virus production rate (p) fixed to the high d/v value (8.6 (PFU/ml) d^{-1} for M-F and 0.9 (PFU/ml) d^{-1} for P-M)) and T_0 estimated (dotted lines) or T_0 fixed to 1×10^7 cells and p estimated (dashed lines). For both viruses, ΔAIC$_c$ between fixing and estimating T_0 were within 2.

Table D: Parameter estimates when estimating the initial number of target cells (T_0) and fixing the virus production rate (p). Estimated population parameters and 95% confidence intervals obtained from fitting the model (Eqs (3)-(6), Main Text) individually to the estimated viral loads from the lungs, trachea, and nasopharynx of mice infected with either the rSeV-luc(M-F*) virus ("M-F") or the rSeV-luc(P-M) virus ("P-M") at low d/v or high d/v.

	**Virus**	**Dose** PFU	**Initial number of target cells, T_0** cell $\times 10^6$	**Virus clearance, c** d^{-1}	**Virus infection, β** $(PFU/ml)^{-1}\ d^{-1}$ $\times 10^{-3}$	**Eclipse phase, k** d^{-1}	**Infected cell clearance, δ_d** $cell^{-1}d^{-1}$ $\times 10^5$	**Half-saturation constant, K_δ** cell $\times 10^3$
**Lung**	**M-F**	**70**	1.0 [0.9-1.1]	14 [9.1-19]	2.2 [1.8-3.0]	3.0 [3.0-3.1]	2.0 [1.8-2.3]	3.1×10^{-1} $[2.3\text{-}3.8]\times10^{-1}$
	**P-M**		1.1 [1.0-1.4]	7.0 [6.1-9.5]	3.4 [2.1-4.4]	3.0 [3.0-3.1]	2.4 [2.2-3.2]	1.2 [1.0-2.1]
**Trachea**	**M-F**	**70**	3.2×10^{-2} $[3.0\times10^{-2}\text{-}4.0\times10^{-2}]$	6.5 [6.1-8.8]	3.1 [2.8-3.4]	3.0 [2.9-3.0]	6.7×10^{-2} $[6.1\times10^{-2}\text{-}7.2\times10^{-2}]$	1.4×10^{-1} $[1.1\times10^{-1}\text{-}1.5\times10^{-1}]$
		**7000**	1.5×10^{-1} $[1.1\times10^{-1}\text{-}1.8\times10^{-1}]$	3.7 [3.4-4.1]	3.5 [3.2-3.8]	3.0 [2.9-3.0]	3.0×10^{-1} $[2.4\times10^{-1}\text{-}4.0\times10^{-1}]$	1.0×10^{-1} $[9.7\times10^{-2}\text{-}1.1\times10^{-1}]$
	**P-M**	**70**	1.1×10^{-1} $[8.0\times10^{-2}\text{-}1.3\times10^{-1}]$	5.0 [4.3-5.6]	5.0 [4.8-5.5]	3.0 [2.9-3.0]	2.4×10^{-1} $[1.6\times10^{-1}\text{-}2.8\times10^{-1}]$	5.2×10^{-1} $[4.7\times10^{-1}\text{-}5.4\times10^{-1}]$
		**7000**	1.2 [1.1-1.3]	4.2 [4.0-4.5]	4.6 [4.3-5.0]	3.0 [2.9-3.0]	2.6 [2.5-2.8]	9.2×10^{-1} $[8.4\times10^{-1}\text{-}9.7\times10^{-1}]$
**Nasopharynx**	**M-F**	**70**	2.5 [2.5-3.0]	3.6 [3.3-4.6]	8.6×10^{-3} $[7.8\times10^{-3}\text{-}1.2\times10^{-2}]$	3.0 [2.9-3.0]	5.5 [4.7-6.0]	8.6×10^{1} $[5.5\times10^{1}\text{-}10.2\times10^{2}]$
		**7000**	4.5 [3.6-5.1]	3.1 [2.6-3.8]	1.5×10^{-2} $[1.1\times10^{-2}\text{-}3.1\times10^{-2}]$	3.0 [2.9-3.0]	8.1 [6.1-8.9]	1.5×10^{2} $[4.7\times10^{1}\text{-}2.0\times10^{2}]$
	**P-M**	**70**	9.0 $[8.5\text{-}1.1\times10^{1}]$	7.1 [5.7-13.2]	4.6×10^{-2} $[3.9\times10^{-2}\text{-}6.0\times10^{-2}]$	3.0 [2.9-3.0]	2.2×10^{1} $[2.1\text{-}3.3]\times10^{1}$	2.6×10^{2} $[2.6\times10^{2}\text{-}3.3\times10^{2}]$
		**7000**	1.6×10^{1} $[1.6\times10^{1}\text{-}2.2\times10^{1}]$	4.9 [4.4-5.6]	7.2×10^{-2} $[3.3\times10^{-2}\text{-}9.1\times10^{-2}]$	3.0 [2.9-3.0]	3.6×10^{1} $[3.5\times10^{1}\text{-}4.2\times10^{1}]$	1.0×10^{2} $[8.6\times10^{1}\text{-}3.4\times10^{2}]$

Dynamics of Target Cells and Infected Cells

Fig D shows the best fit solutions of the model (Eqs (3)-(6)) for target cells (T_0) and infected cells (I_1, I_2). Virus is plotted in Fig 2

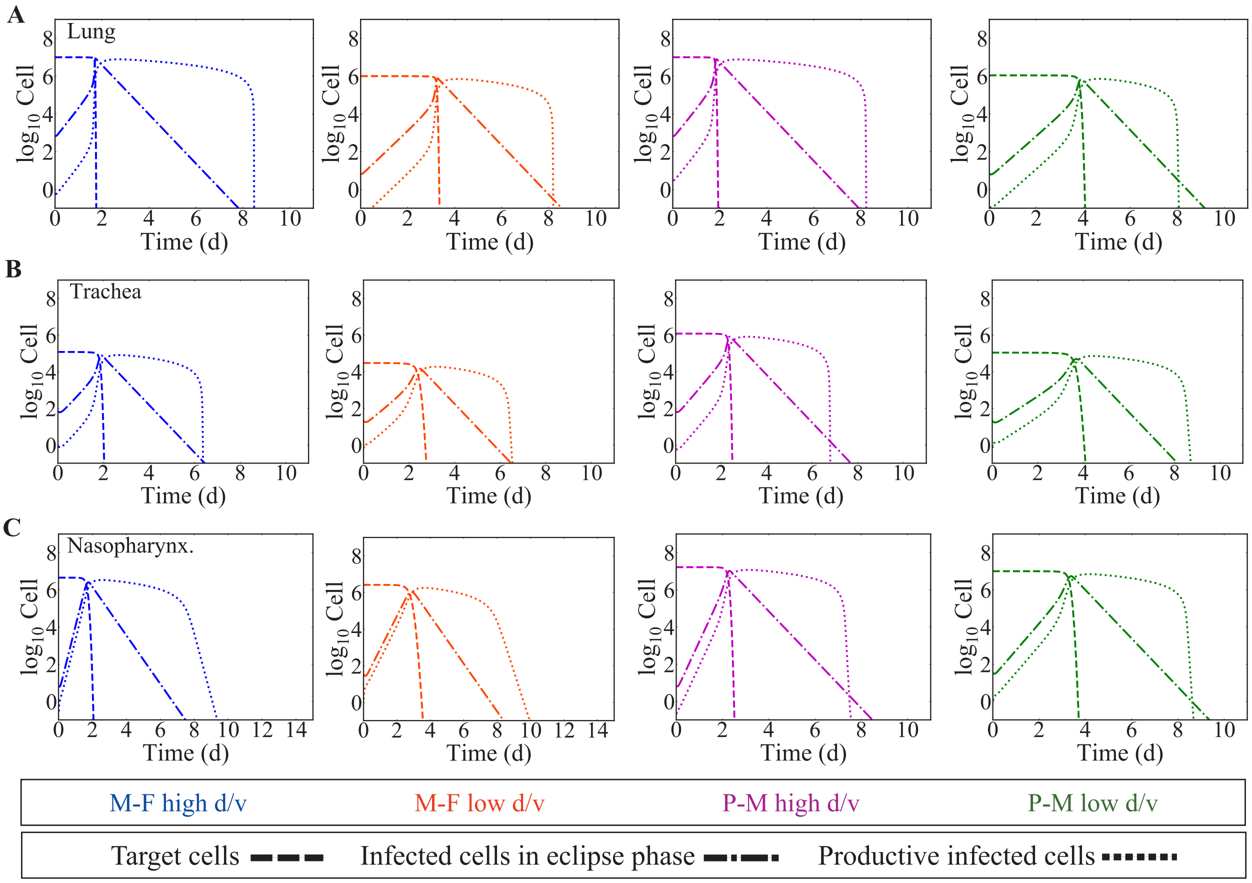

Figure D: Model solutions for target, eclipse and productive infected cell dynamics. (A-C) Best fit solutions of the model (Eqs (3)-(6)) using the population estimates (Table 2, Main Text) for target cells (T; dashed line), infected cells in eclipse phase (I_1; dash-dotted line) and productive infected cells (I_2; dotted line) for data from the lung (Panel A; squares), trachea (Panel B; diamonds) and nasopharynx (Panel C; circles) of mice infected with rSeV-luc(M-F*) ("M-F"; black) at high d/v (solid blue line) or low d/v (dashed orange line) or with rSeV-luc(P-M) ("P-M"; white) at high d/v (solid magenta line) or low d/v (dashed green line).

Additional Parameter Ensembles

Figs E–K show parameter histograms and ensembles obtained from fitting the model (Eqs (3)-(6)) individually to the estimated viral loads from the lungs, trachea, and nasopharynx of mice infected with high d/v or low d/v of the rSeV-luc(M-F*) virus or the rSeV-luc(P-M) virus. All other parameter histograms and ensembles are in Figs 3- 4 (Main Text).

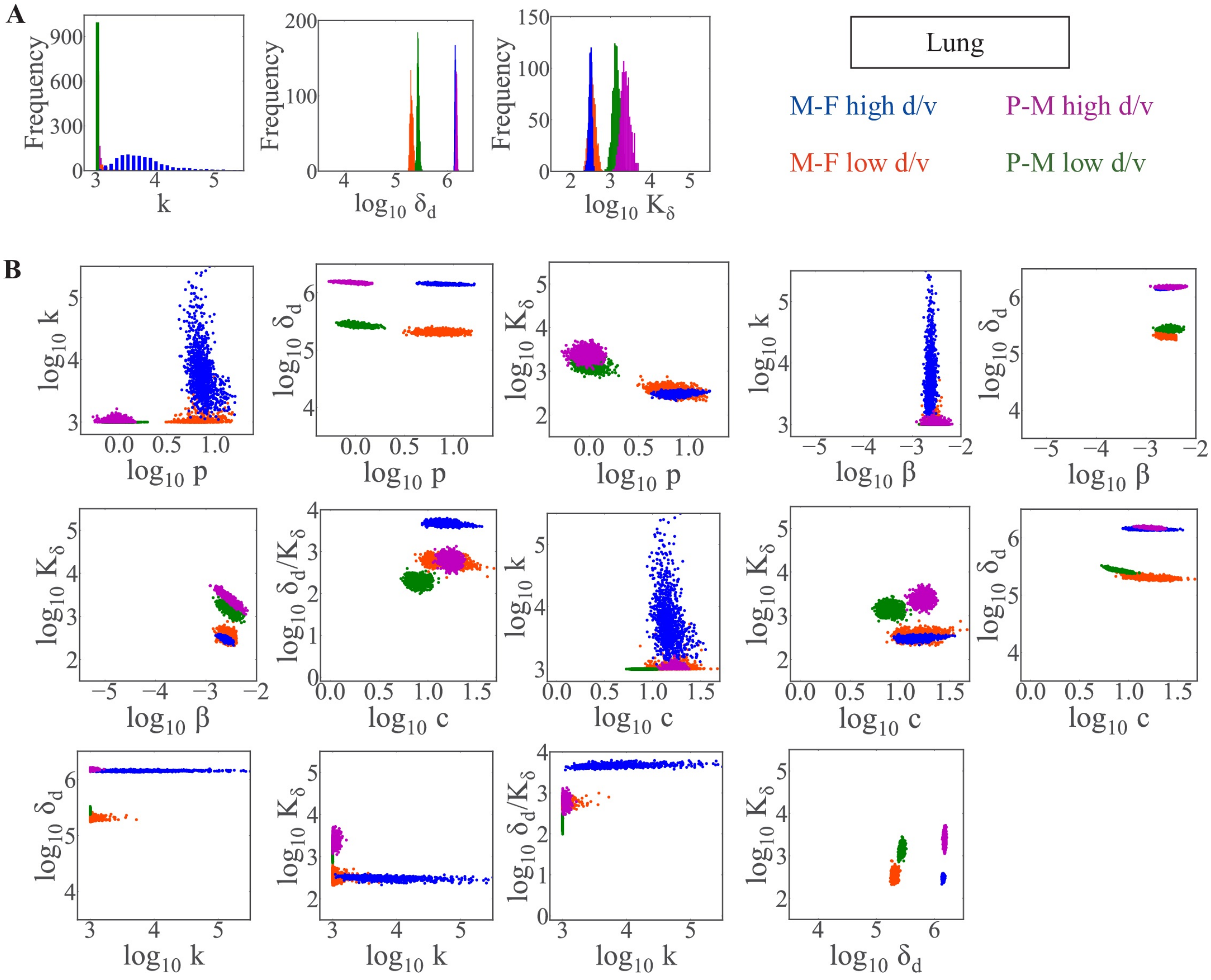

Figure E: Parameter histograms and ensembles for infection in the lung. Parameter histograms (Panel A) and ensembles (Panel B) resulting from fitting the model (Eqs (3)-(6)) to the estimated viral loads from the lungs of mice infected with the rSeV-luc(M-F*) virus ("M-F") at high d/v (blue) or low d/v (orange) or the rSeV-luc(P-M) virus ("P-M") at high d/v (magenta) or low d/v (green). Parameters shown are virus production (p), virus clearance (c), virus infection (β), eclipse phase transition rate (k), and infected cell clearance (δ_d, K_δ). Additional plots are in Fig 3 (Main Text).

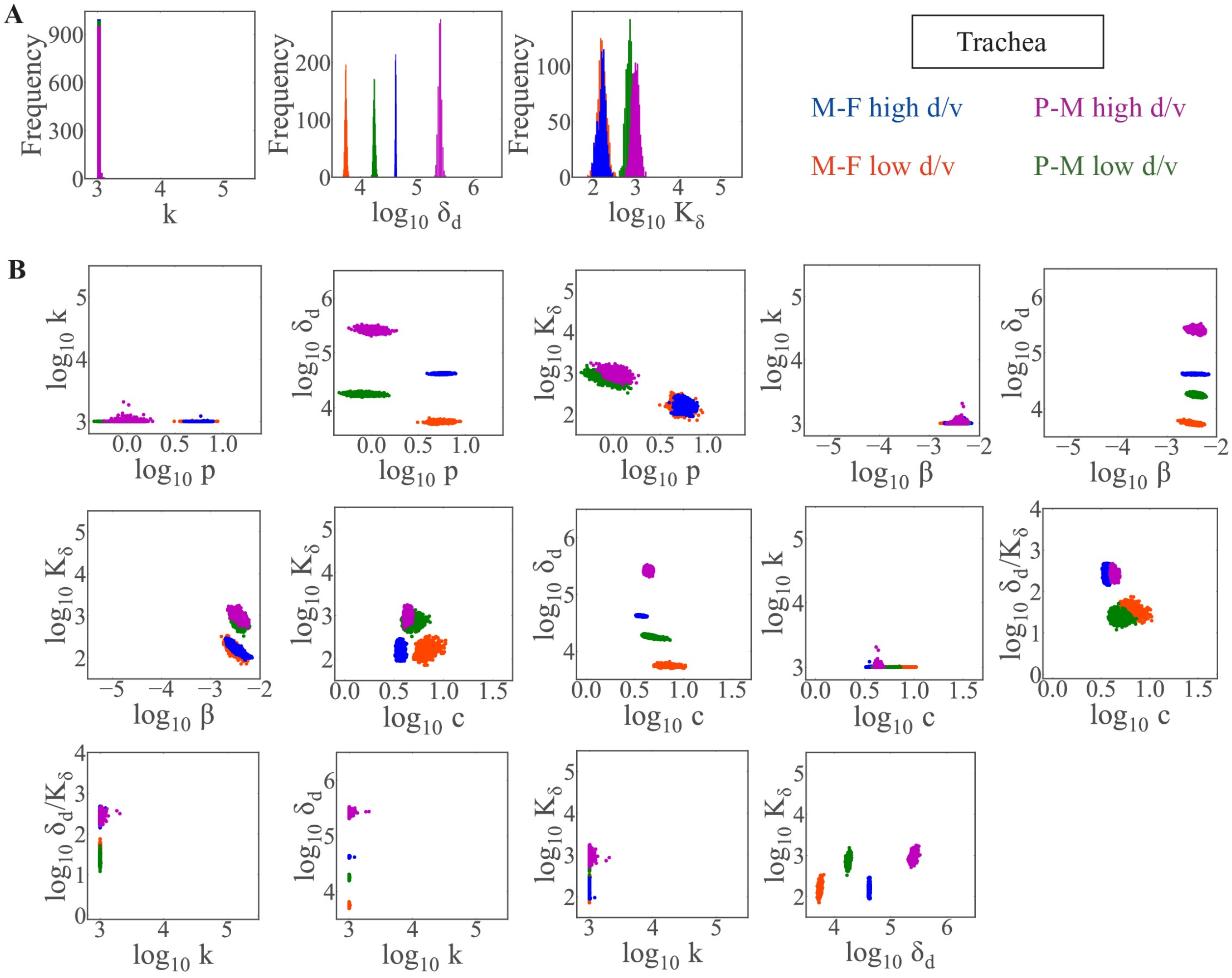

Figure F: Parameter histograms and ensembles for infection in the trachea. Parameter histograms (Panel A) and ensembles (Panel B) resulting from fitting the model (Eqs (3)-(6)) to the estimated viral loads from the trachea of mice infected with the rSeV-luc(M-F*) virus ("M-F") at high d/v (blue) or low d/v (orange) or the rSeV-luc(P-M) virus ("P-M") at high d/v (magenta) or low d/v (green). Parameters shown are virus production (p), virus clearance (c), virus infection (β), eclipse phase transition rate (k), and infected cell clearance (δ_d, K_δ). Additional plots are in Fig 3 (Main Text).

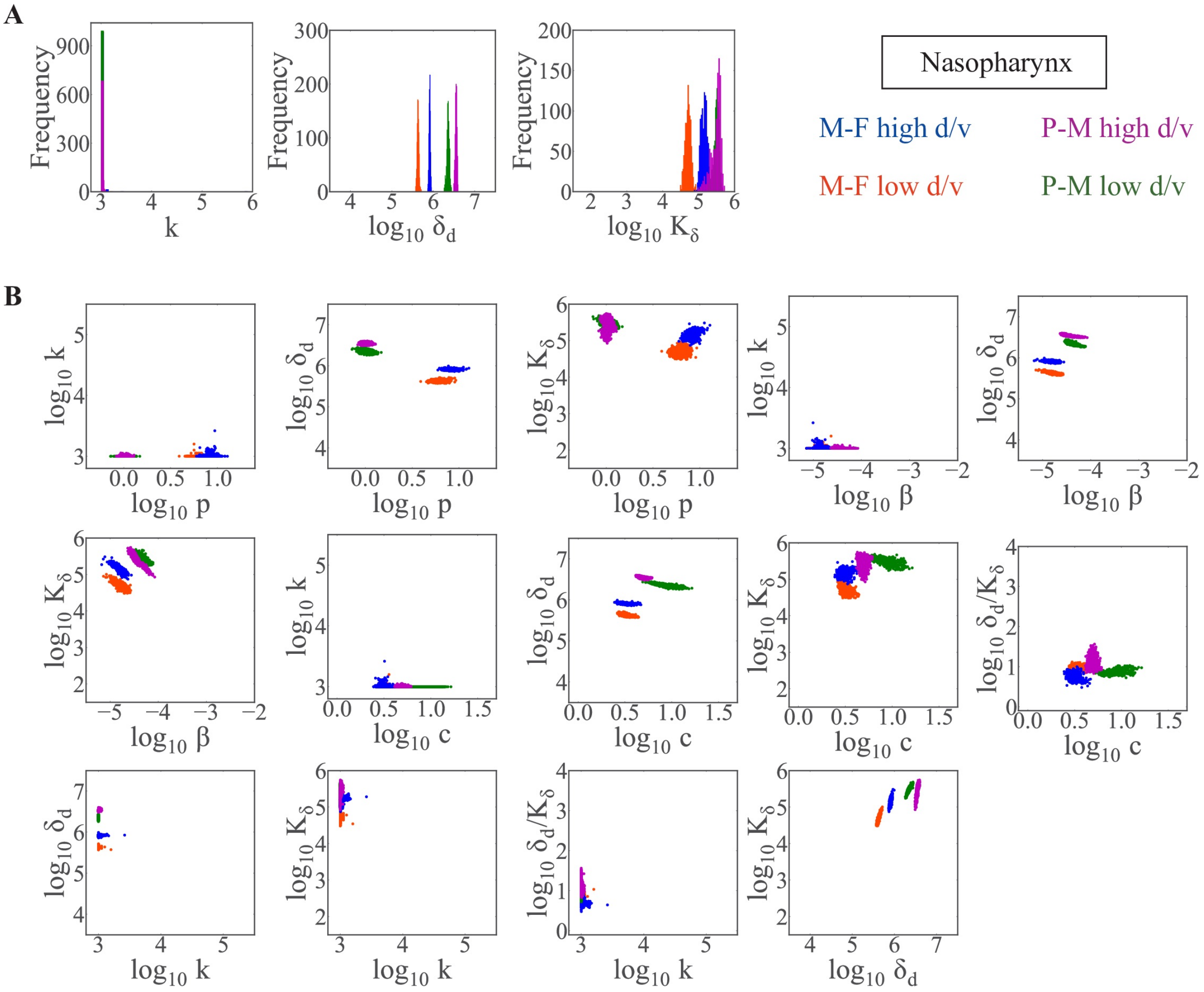

Figure G: Parameter histograms and ensembles for infection in the nasopharynx. Parameter histograms (Panel A) and ensembles (Panel B) resulting from fitting the model (Eqs (3)-(6)) to the estimated viral loads from the nasopharynx of mice infected with the rSeV-luc(M-F*) virus ("M-F") at high d/v (blue) or low d/v (orange) or the rSeV-luc(P-M) virus ("P-M") at high d/v (magenta) or low d/v (green). Parameters shown are virus production (p), virus clearance (c), virus infection (β), eclipse phase transition rate (k), and infected cell clearance (δ_d, K_δ). Additional plots are in Fig 3 (Main Text).

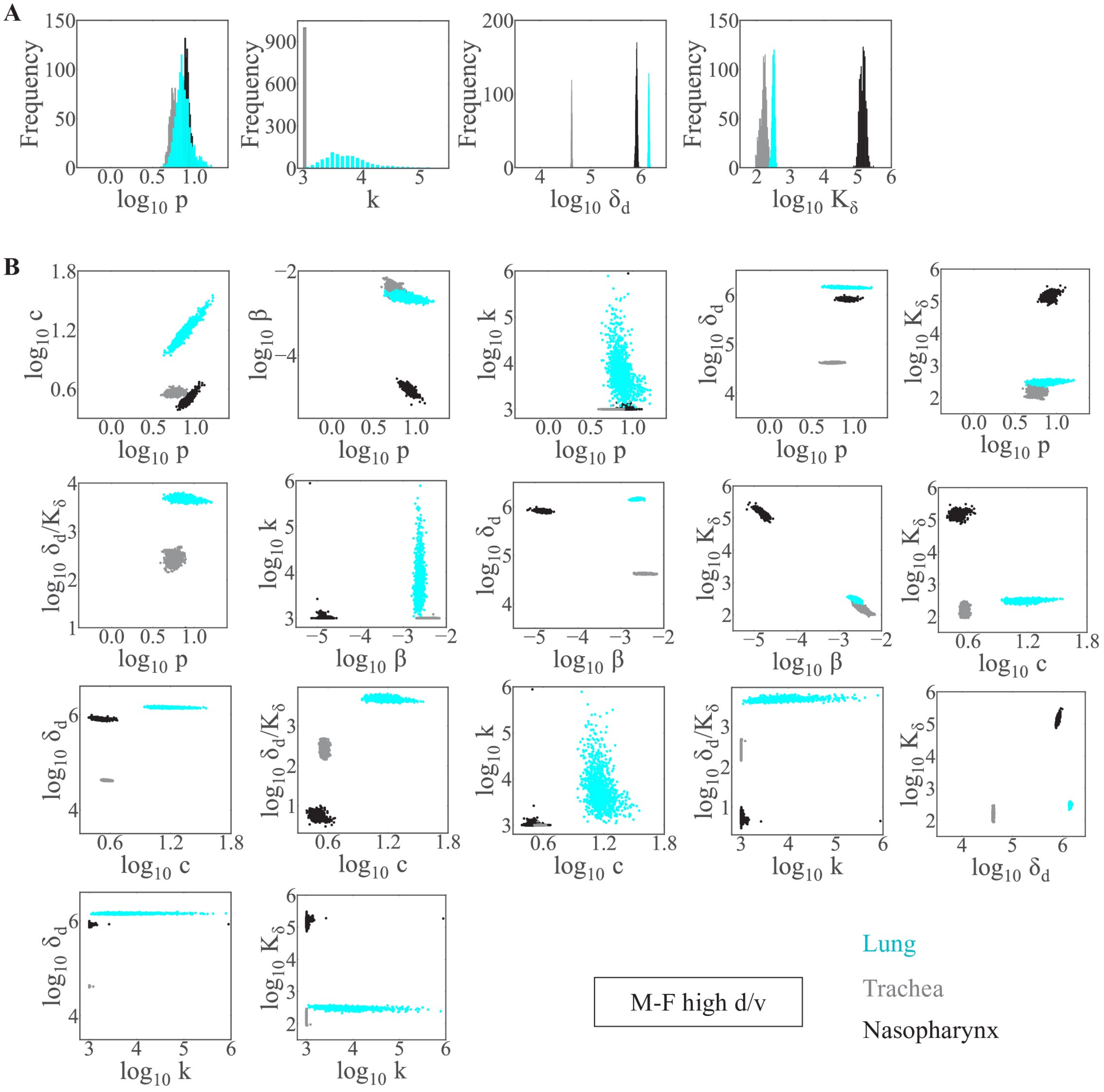

Figure H: Parameter histograms and ensembles for the M-F virus at high d/v. Parameter histograms (Panel A) and ensembles (Panel B) resulting from fitting the model (Eqs (3)-(6)) individually to the estimated viral loads from the lungs (cyan), trachea (gray), and nasopharynx (black) of mice infected with the rSeV-luc(M-F*) virus ("M-F") at high d/v. Parameters shown are virus production (p), virus clearance (c), virus infection (β), eclipse phase transition rate (k), and infected cell clearance (δ_d, K_δ). Additional plots are in Fig 4 (Main Text).

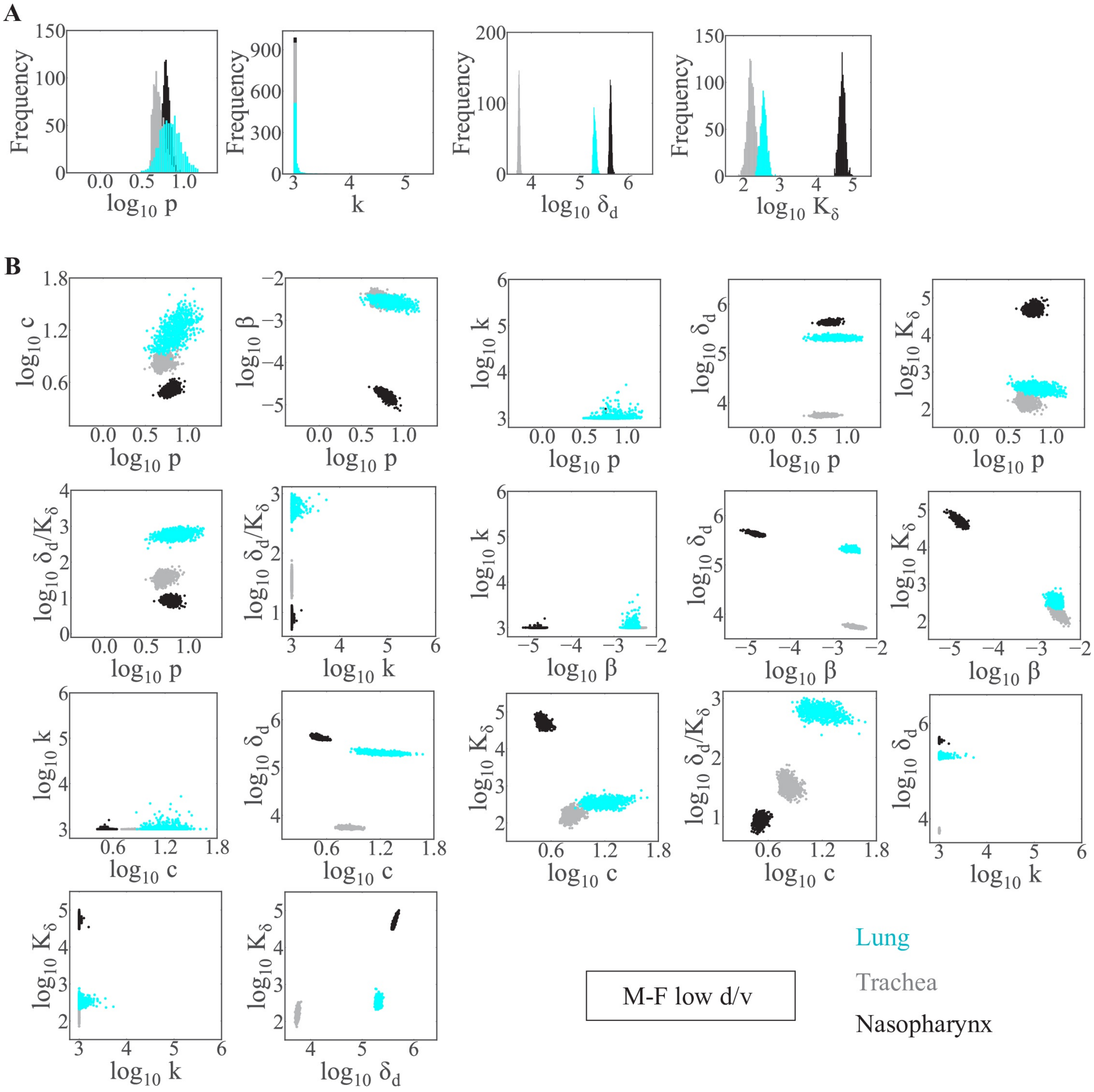

Figure I: Parameter histograms and ensembles for the M-F virus at low d/v. Parameter histograms (Panel A) and ensembles (Panel B) resulting from fitting the model (Eqs (3)-(6)) individually to the estimated viral loads from the lungs (cyan), trachea (gray), and nasopharynx (black) of mice infected with the rSeV-luc(M-F*) virus ("M-F") at low d/v. Parameters shown are virus production (p), virus clearance (c), virus infection (β), eclipse phase transition rate (k), and infected cell clearance (δ_d, K_δ). Additional plots are in Fig 4 (Main Text).

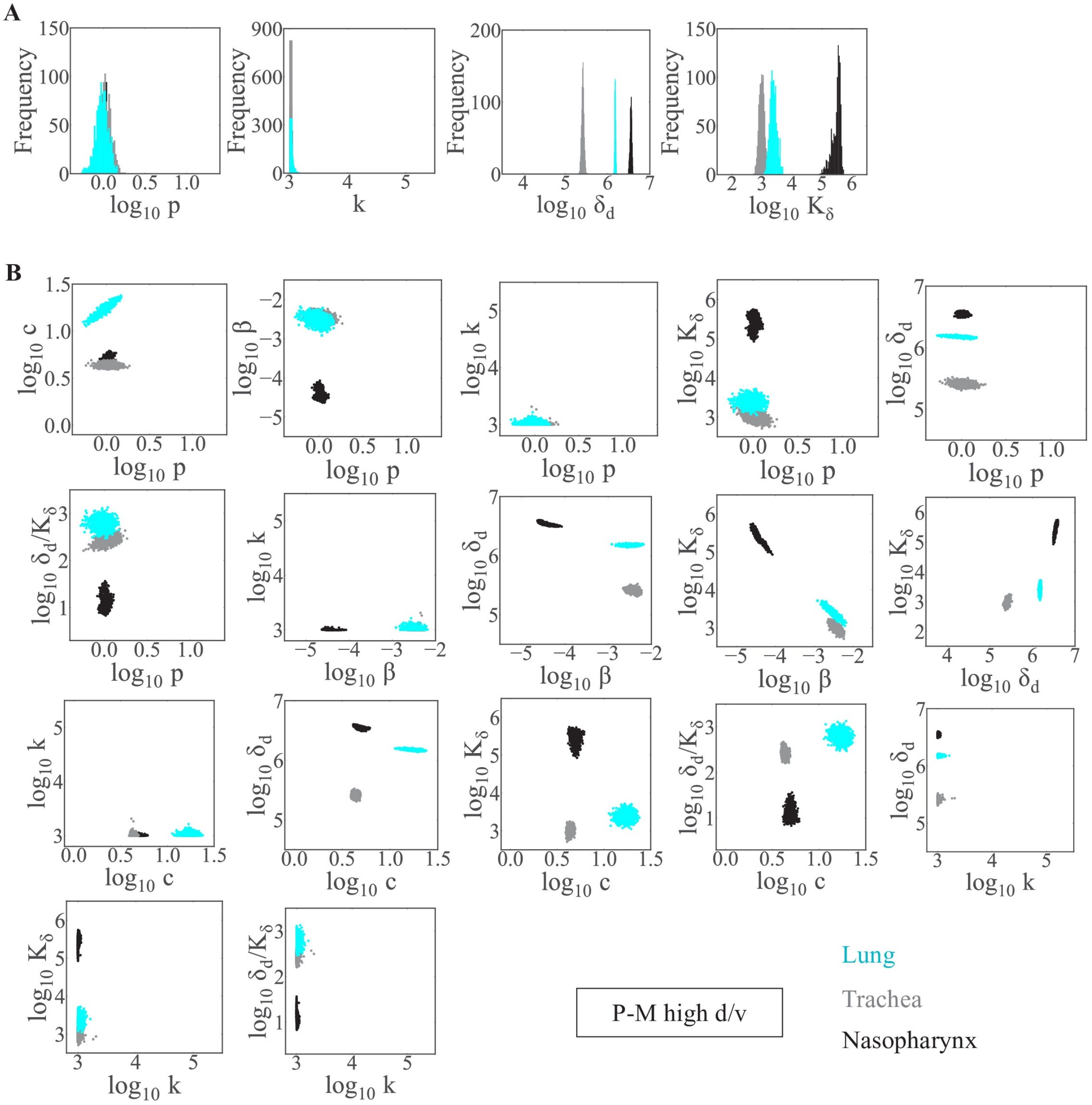

Figure J: Parameter histograms and ensembles for the P-M virus at high d/v. Parameter histograms (Panel A) and ensembles (Panel B) resulting from fitting the model (Eqs (3)-(6)) individually to the estimated viral loads from the lungs (cyan), trachea (gray), and nasopharynx (black) of mice infected with the rSeV-luc(P-M) virus ("P-M") at high d/v. Parameters shown are virus production (p), virus clearance (c), virus infection (β), eclipse phase transition rate (k), and infected cell clearance (δ_d, K_δ). Additional plots are in Fig 4 (Main Text).

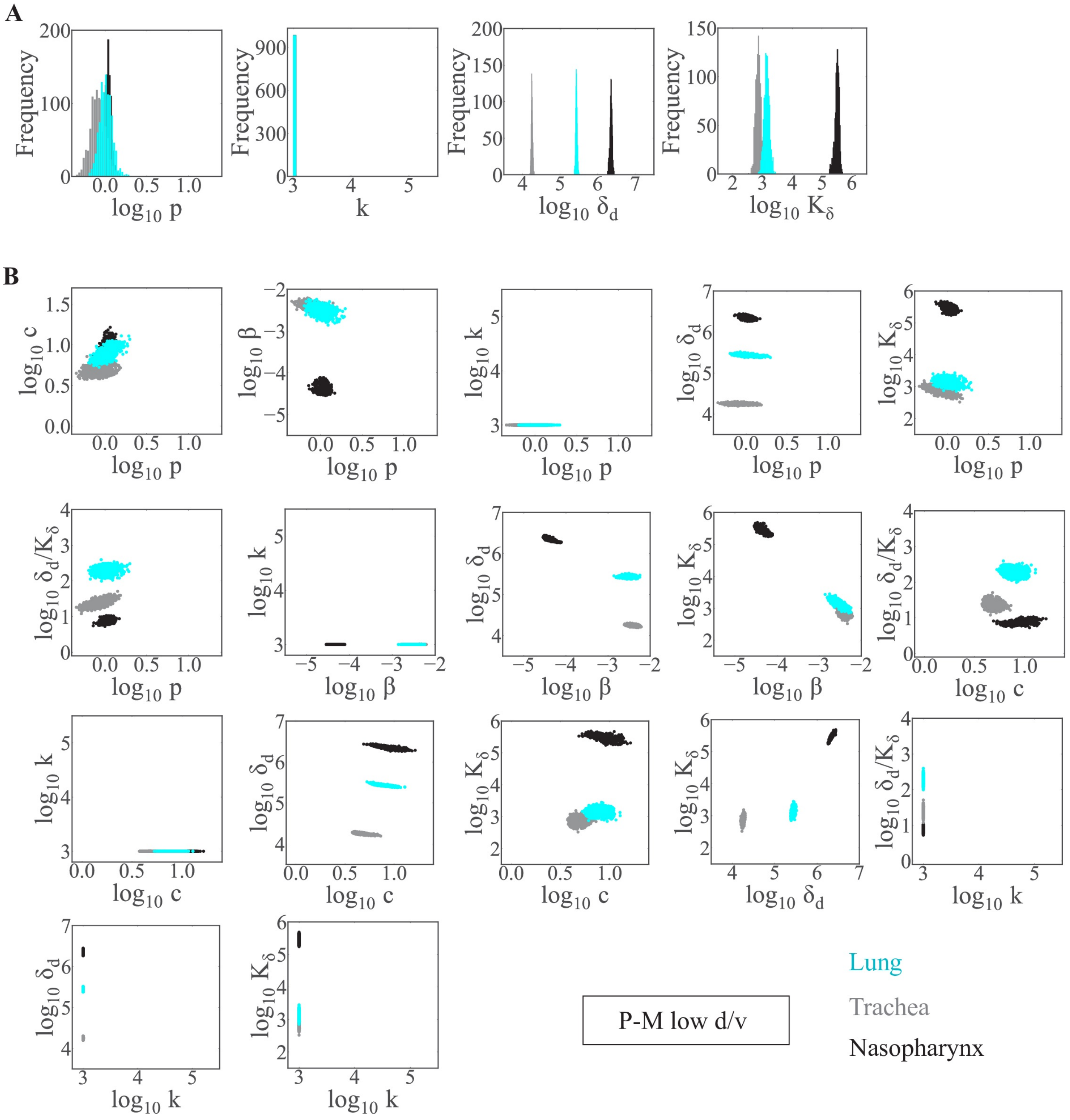

Figure K: Parameter histograms and ensembles for the P-M virus at low d/v. Parameter histograms (Panel A) and ensembles (Panel B) resulting from fitting the model (Eqs (3)-(6)) individually to the estimated viral loads from the lungs (cyan), trachea (gray), and nasopharynx (black) of mice infected with the rSeV-luc(P-M) virus ("P-M") at low d/v. Parameters shown are virus production (p), virus clearance (c), virus infection (β), eclipse phase transition rate (k), and infected cell clearance (δ_d, K_δ). Additional plots are in Fig 4 (Main Text).

Individual Fits

Individual fits were obtained by fitting the model (Eqs (3)-(6)) to data from each individual in each infection group (Figs L-W). Random effects were considered for all the parameters and the standard deviation (ω) values were estimated (Table E).

Table E: Estimates of the standard deviation of random effect parameters. Estimated standard deviation and 95% confidence intervals obtained from fitting the model (Eqs (3)-(6), Main Text) to the estimated viral loads from the lung, trachea, and nasopharynx of each individual mice infected with either the rSeV-luc(M-F*) virus ("M-F") or the rSeV-luc(P-M) virus ("P-M") at high d/v or a low d/v.

	**Virus**	**Dose** PFU	**Virus production,** ω_p (PFU/ml) cell^{-1} d^{-1}	**Virus clearance,** ω_c d^{-1}	**Virus infection,** ω_β (PFU/ml)$^{-1}$ d^{-1}	**Eclipse phase,** ω_k d^{-1}	**Infected cell clearance,** ω_δ d^{-1}	**Half-saturation constant,** ω_{K_δ} cells
**Lung**	**M-F**	**70**	0.02 [0.01–0.4]	0.04 [0.01–0.3]	0.08 [0.05–1.0]	0.46 [0.4–4.8]	0.17 [0.05–0.3]	0.09 [0.03–0.6]
		**7000**	0.18 [0.02–0.63]	0.18 [0.03–0.69]	0.06 [0.01–0.34]	1.41 [0.20–1.66]	0.07 [0.08–0.35]	0.46 [0.13–1.04]
	**P-M**	**70**	0.01 [0–0.04]	0.04 [0.01–0.07]	0.02 [0–0.04]	5.86 [3.5–22]	0.12 [0.04–0.17]	0.02 [0–0.04]
		**7000**	0.08 [0.01–0.18]	0.21 [0.02–0.17]	0.10 [0.02–0.25]	0.88 [0.36–2.98]	0.14 [0.10–0.18]	0.23 [0.01–0.31]
**Trachea**	**M-F**	**70**	0.01 [0.01–0.04]	0.01 [0.01–0.05]	0.01 [0.01–0.03]	6.18 [1.8–10]	0.02 [0.01–0.03]	0.01 [0.01–0.03]
		**7000**	0.01 [0.00–0.04]	0.10 [0.01–0.12]	0.36 [0.01–0.41]	10.3 [3.73–37]	0.01 [0.00–0.02]	0.02 [0.02–0.71]
	**P-M**	**70**	0.01 [0.00–0.02]	0.02 [0.01–0.08]	0.02 [0.01–0.04]	3.07 [1.7–11]	0.04 [0.01–0.07]	0.01 [0.01–0.03]
		**7000**	0.04 [0.00–0.06]	0.05 [0.00–0.09]	0.04 [0.00–0.04]	4.25 [0.55–4.25]	0.01 [0.00–0.02]	0.01 [0.00–0.05]
**Nasopharynx**	**M-F**	**70**	0.04 [0.02–0.05]	0.13 [0.04–0.13]	0.03 [0.02–0.05]	2.54 [1.6–3.01]	0.03 [0.02–0.13]	0.05 [0.03–0.1]
		**7000**	0.05 [0.01–0.08]	0.04 [0.01–0.27]	0.14 [0.01–0.23]	0.97 [0.71–4.68]	0.05 [0.01–0.10]	0.04 [0.03–0.13]
	**P-M**	**70**	0.03 [0.02–0.05]	0.14 [0.03–0.21]	0.02 [0.01–0.04]	0.47 [0.4–5.2]	0.02 [0.01–0.03]	0.10 [0.02–0.23]
		**7000**	0.08 [0.01–0.11]	0.04 [0.01–0.10]	0.03 [0.01–0.08]	0.63 [0.47–4.64]	0.02 [0.00–0.06]	0.11 [0.03–0.22]

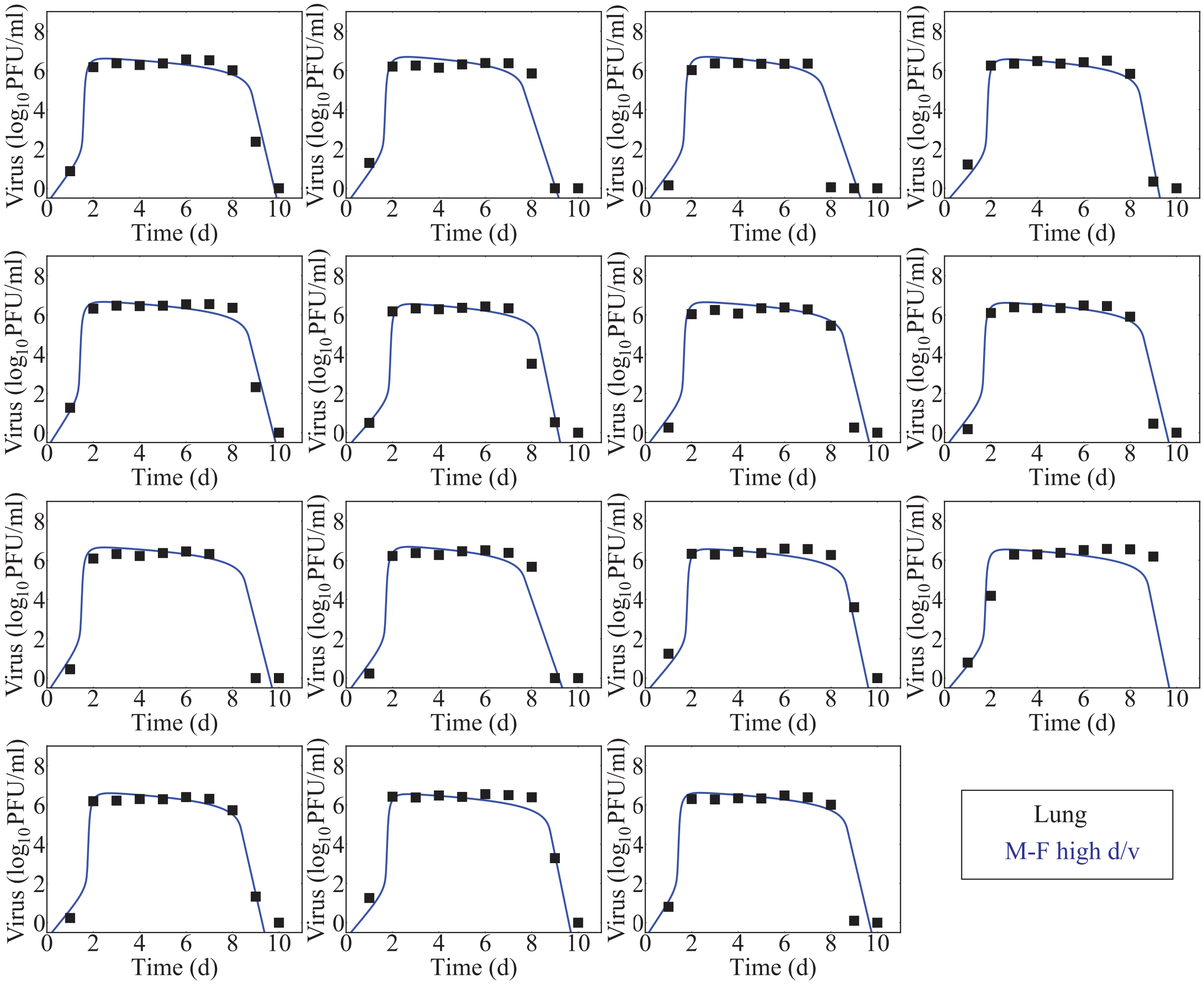

Figure L: Individual fits for infection with rSeV-luc(M-F*) at high d/v in the lung. Fit of the model (Eqs (3)-(6), Main Text) to the estimated viral loads from the lungs of individual mice infected with rSeV-luc(M-F*) (black squares) at high d/v.

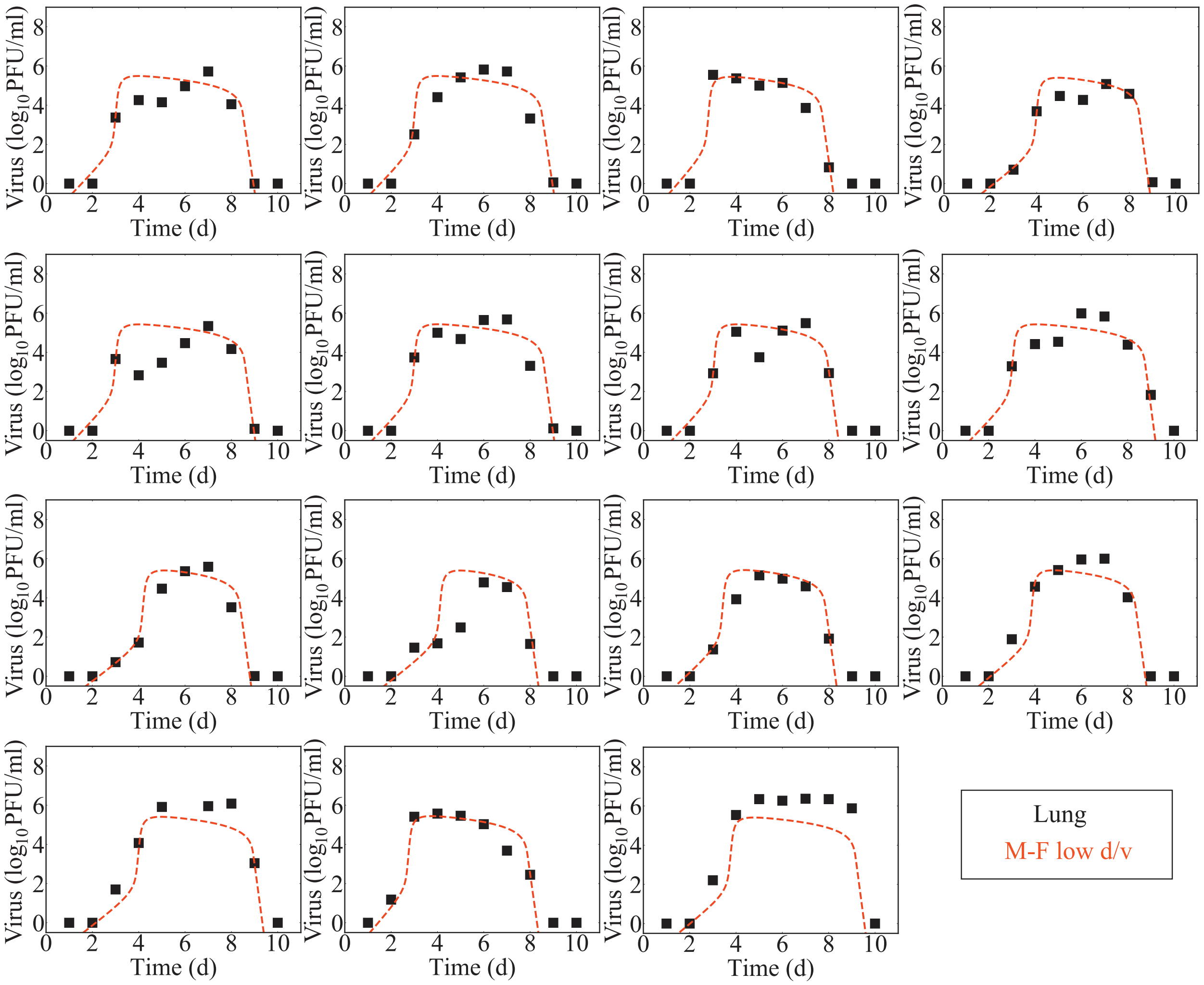

Figure M: Individual fits for infection with the rSeV-luc(M-F*) at low d/v in the lung. Fit of the model (Eqs (3)-(6), Main Text) to the estimated viral loads from the lungs of individual mice infected with rSeV-luc(M-F*) (black squares) at low d/v.

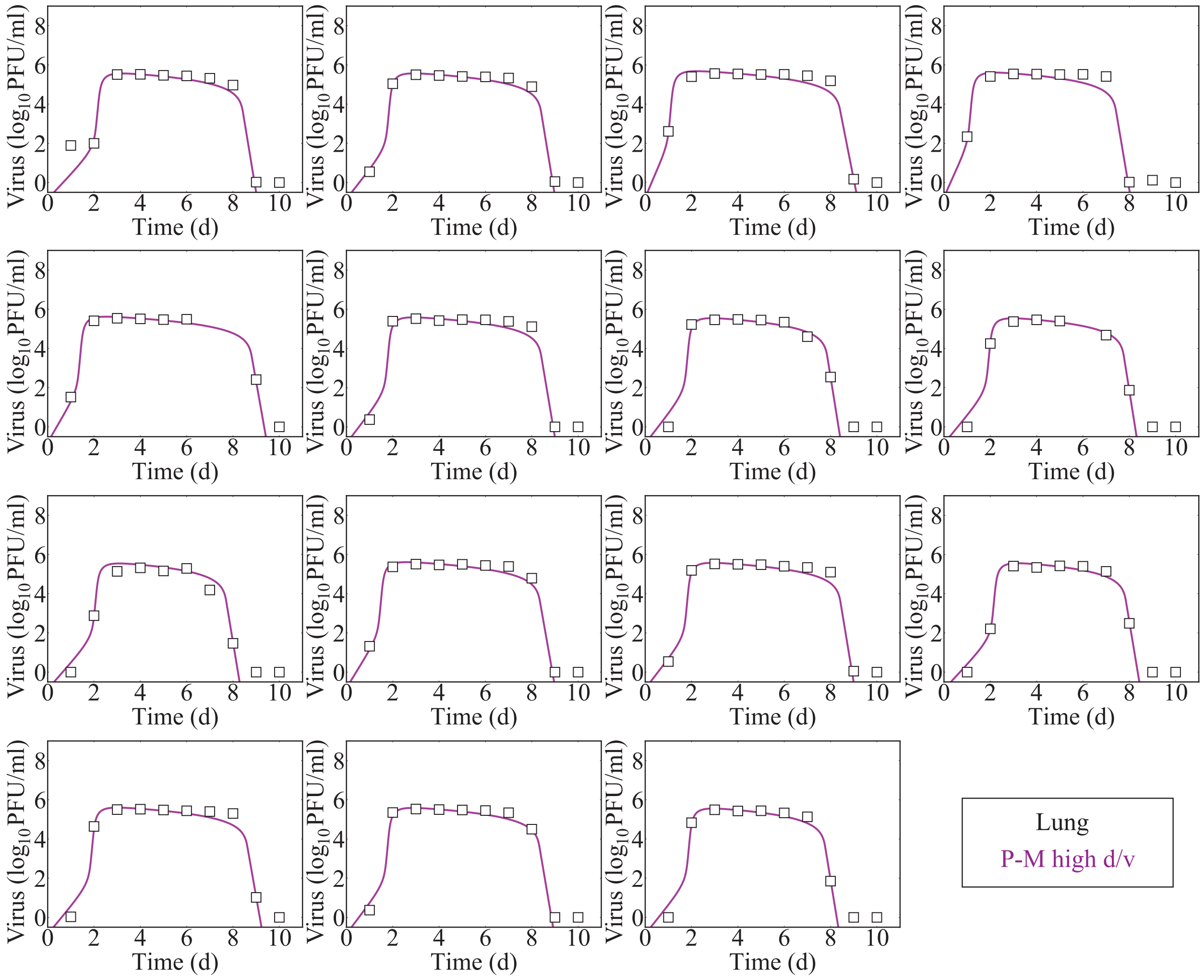

Figure N: Individual fits for infection with the rSeV-luc(P-M) at high d/v in the lung. Fit of the model (Eqs (3)-(6), Main Text) to the estimated viral loads from the lungs of individual mice infected with rSeV-luc(P-M) (white circles) at high d/v.

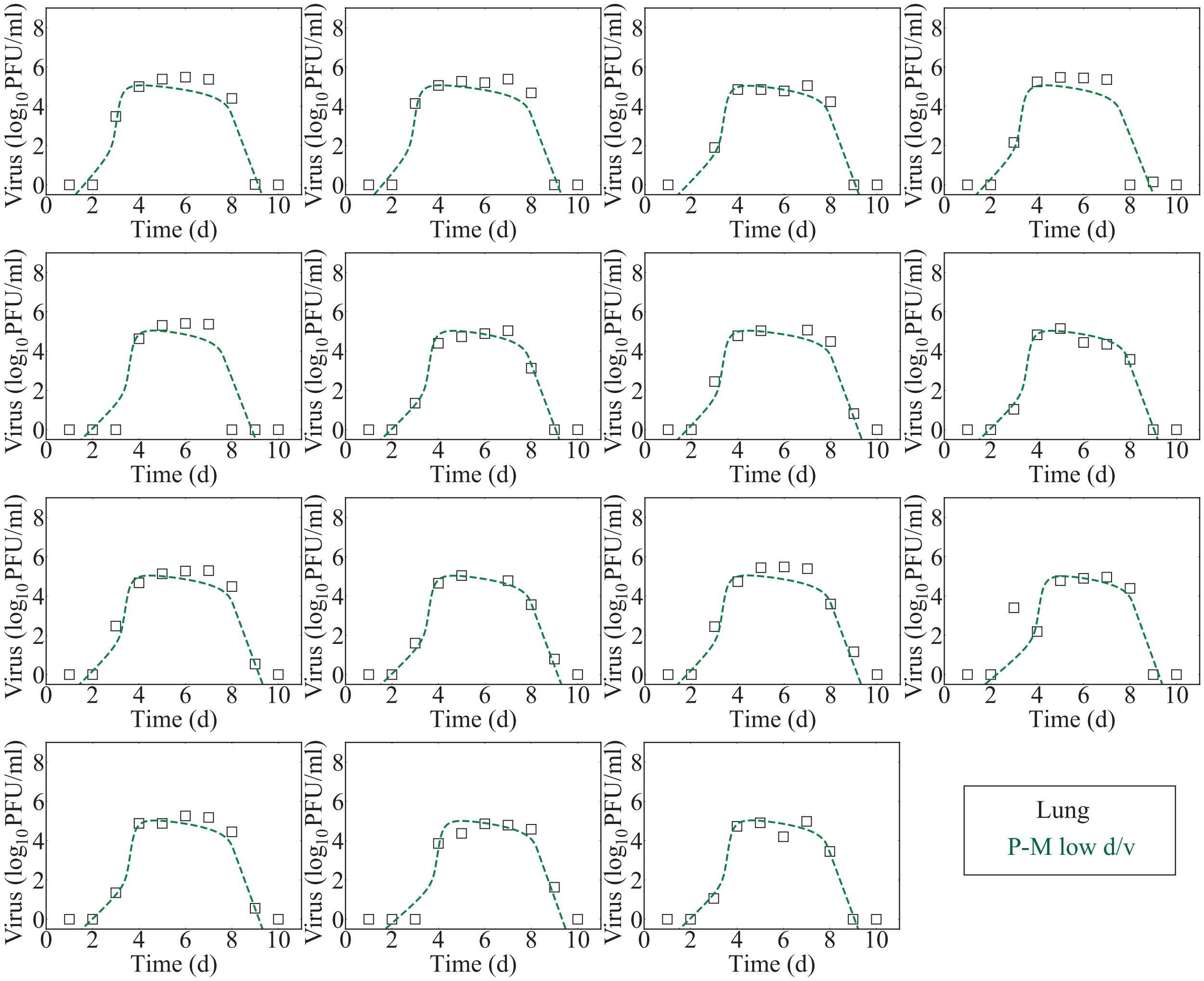

Figure O: Individual fits for the rSeV-luc(P-M) at low d/v in the lung. Fit of the model (Eqs (3)-(6), Main Text) to the estimated viral loads from the lungs of individual mice infected with rSeV-luc(P-M) (white circles) at low d/v.

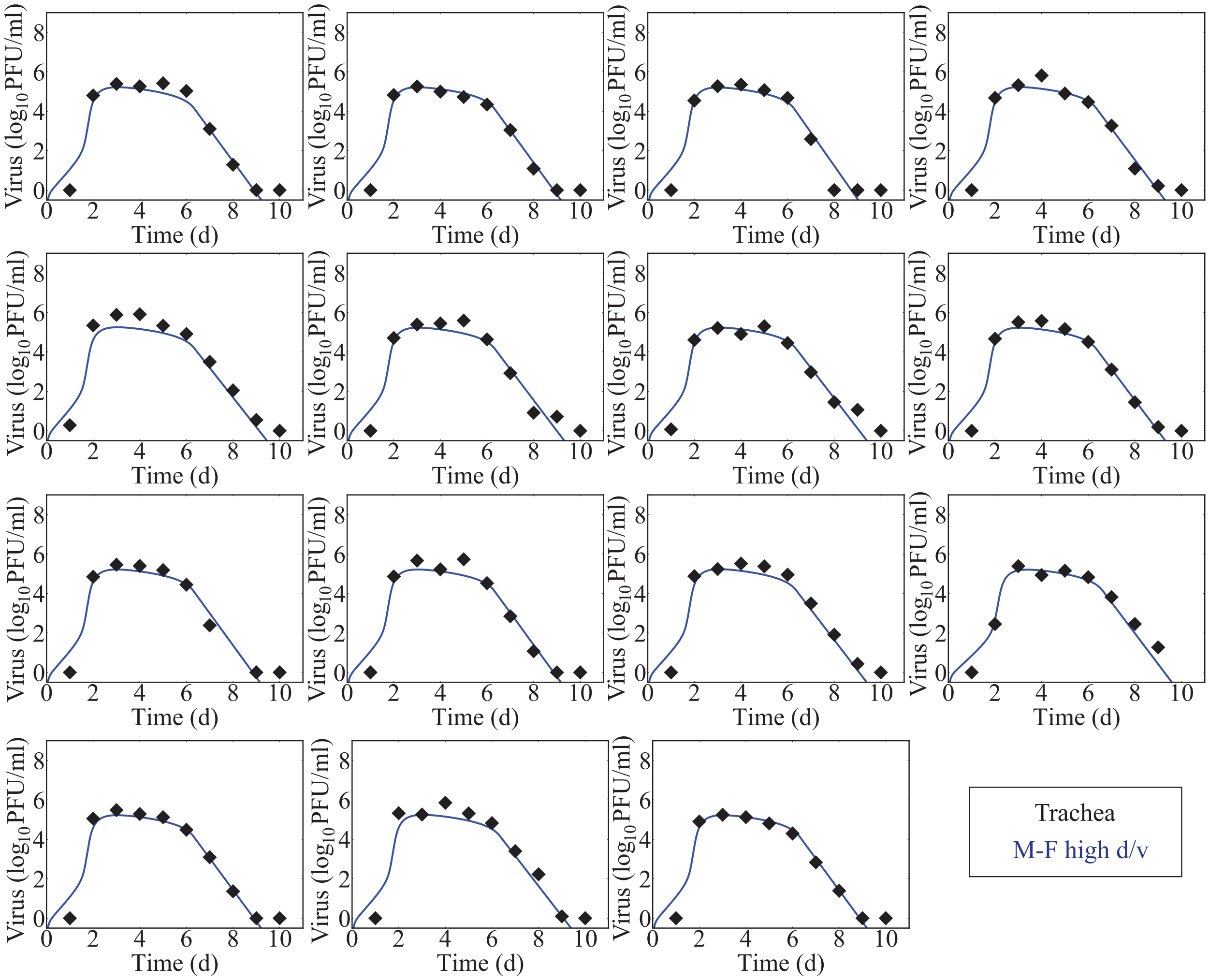

Figure P: Individual fits for the rSeV-luc(M-F*) at high d/v in the trachea. Fit of the model (Eqs (3)-(6), Main Text) to the estimated viral loads from the trachea of individual mice infected with rSeV-luc(M-F*) (black circles) at high d/v.

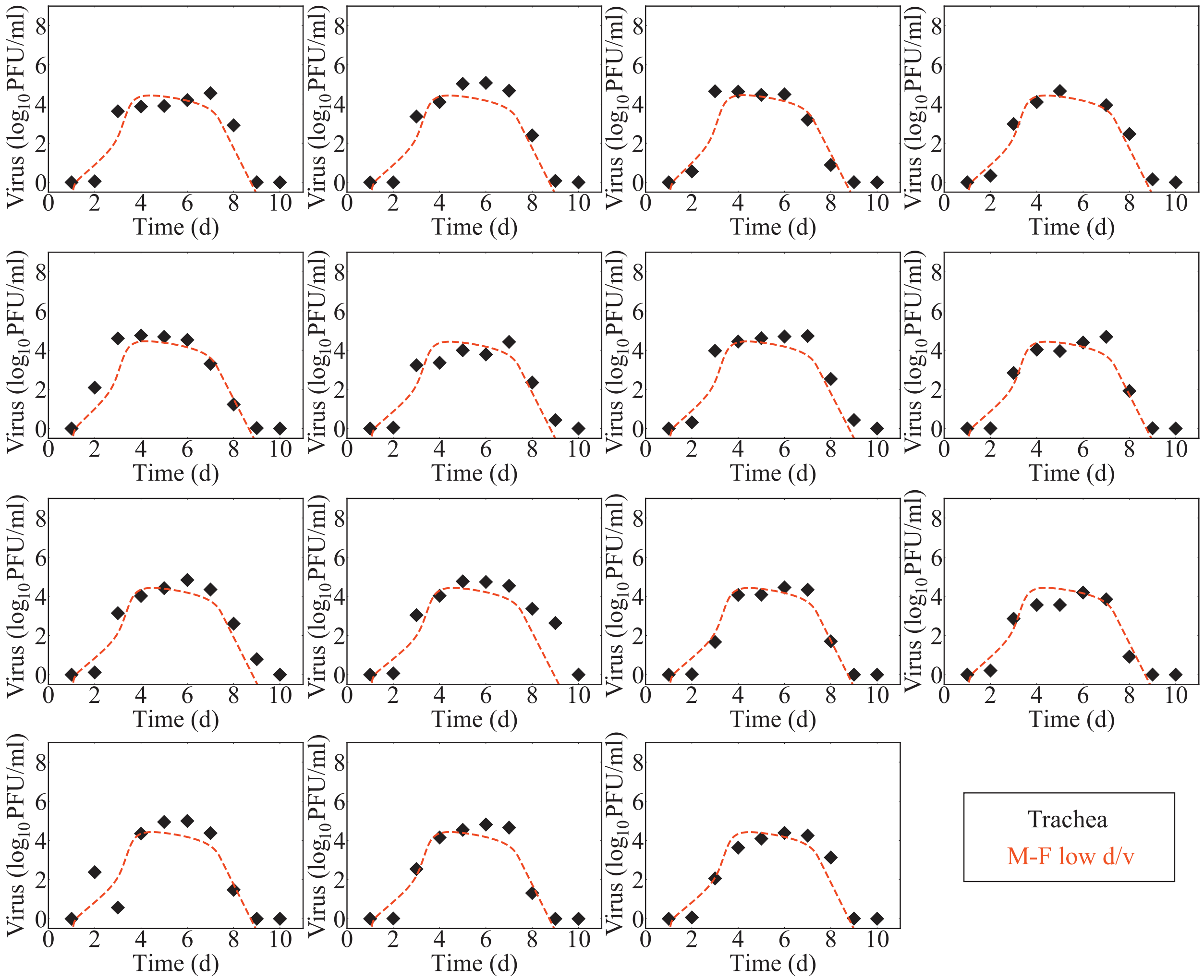

Figure Q: Individual fits for the rSeV-luc(M-F*) at low d/v in the trachea. Fit of the model (Eqs (3)-(6), Main Text) to the estimated viral loads from the trachea of individual mice infected with rSeV-luc(M-F*) (black circles) at low d/v.

s

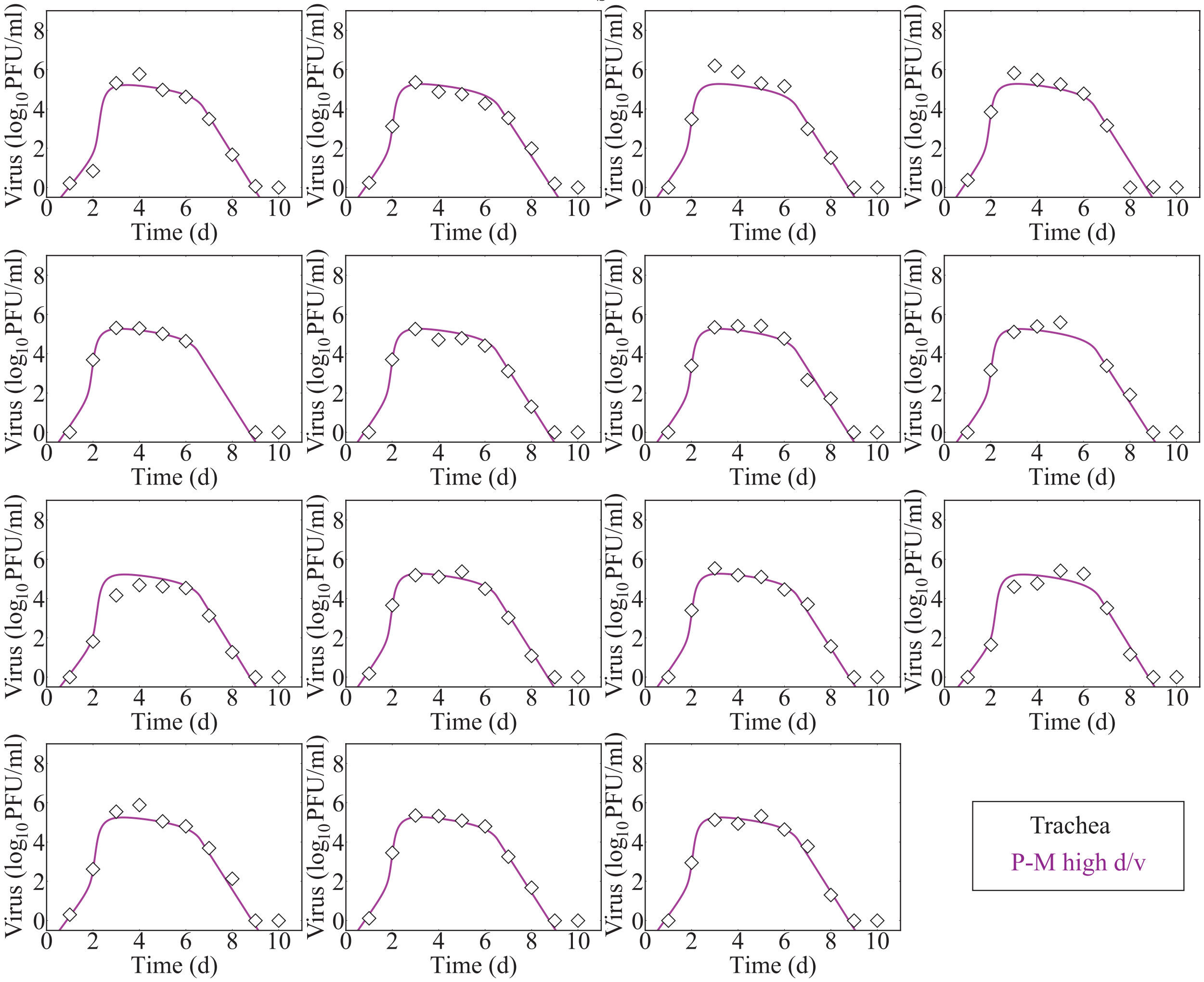

Figure R: Individual fits for the rSeV-luc(P-M) at high d/v in the trachea. Fit of the model (Eqs (3)-(6), Main Text) to the estimated viral loads from the trachea of individual mice infected with rSeV-luc(P-M) (white circles) at high d/v.

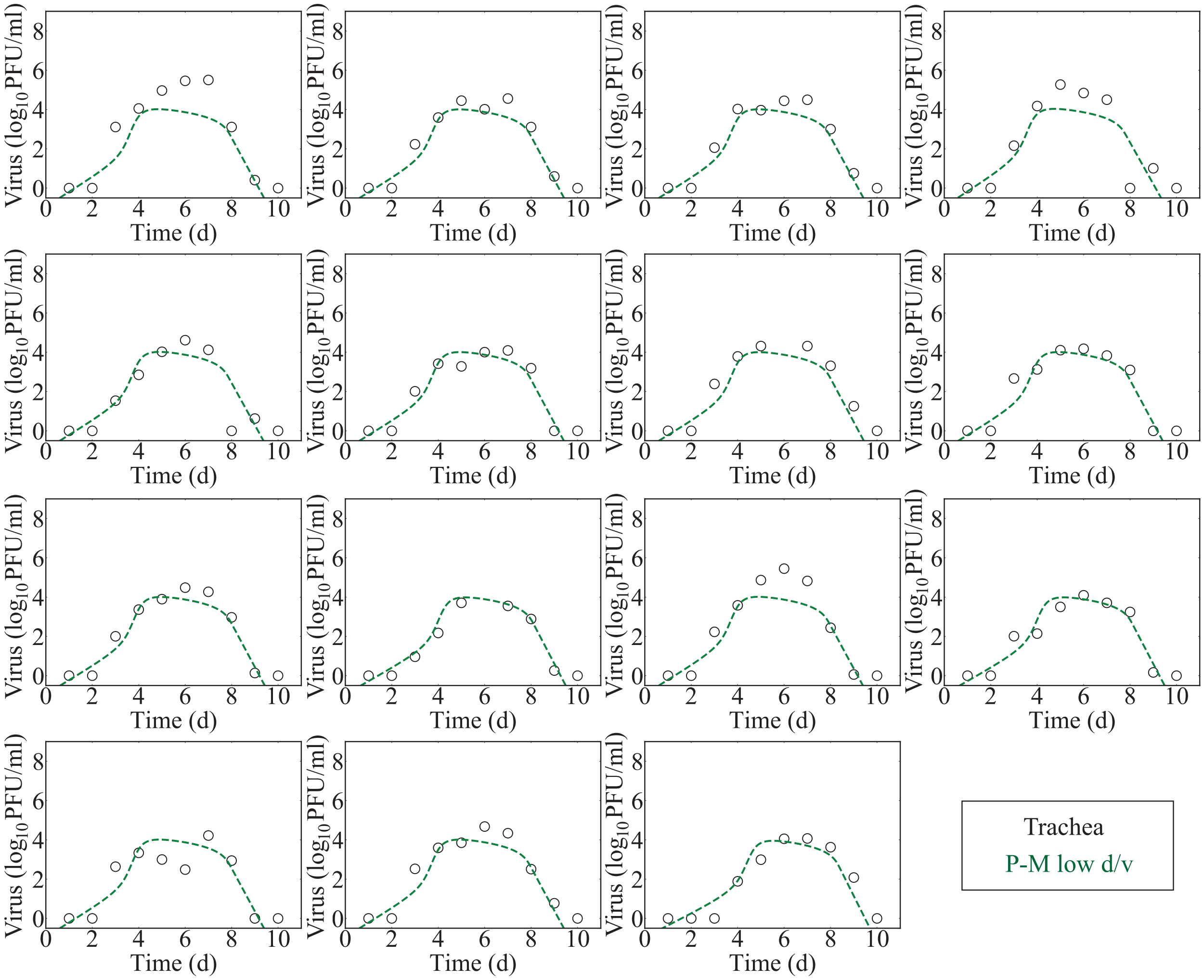

Figure S: Individual fits for the rSeV-luc(P-M) at low d/v in the trachea. Fit of the model (Eqs (3)-(6), Main Text) to the estimated viral loads from the trachea of individual mice infected with rSeV-luc(P-M) (white circles) at low d/v.

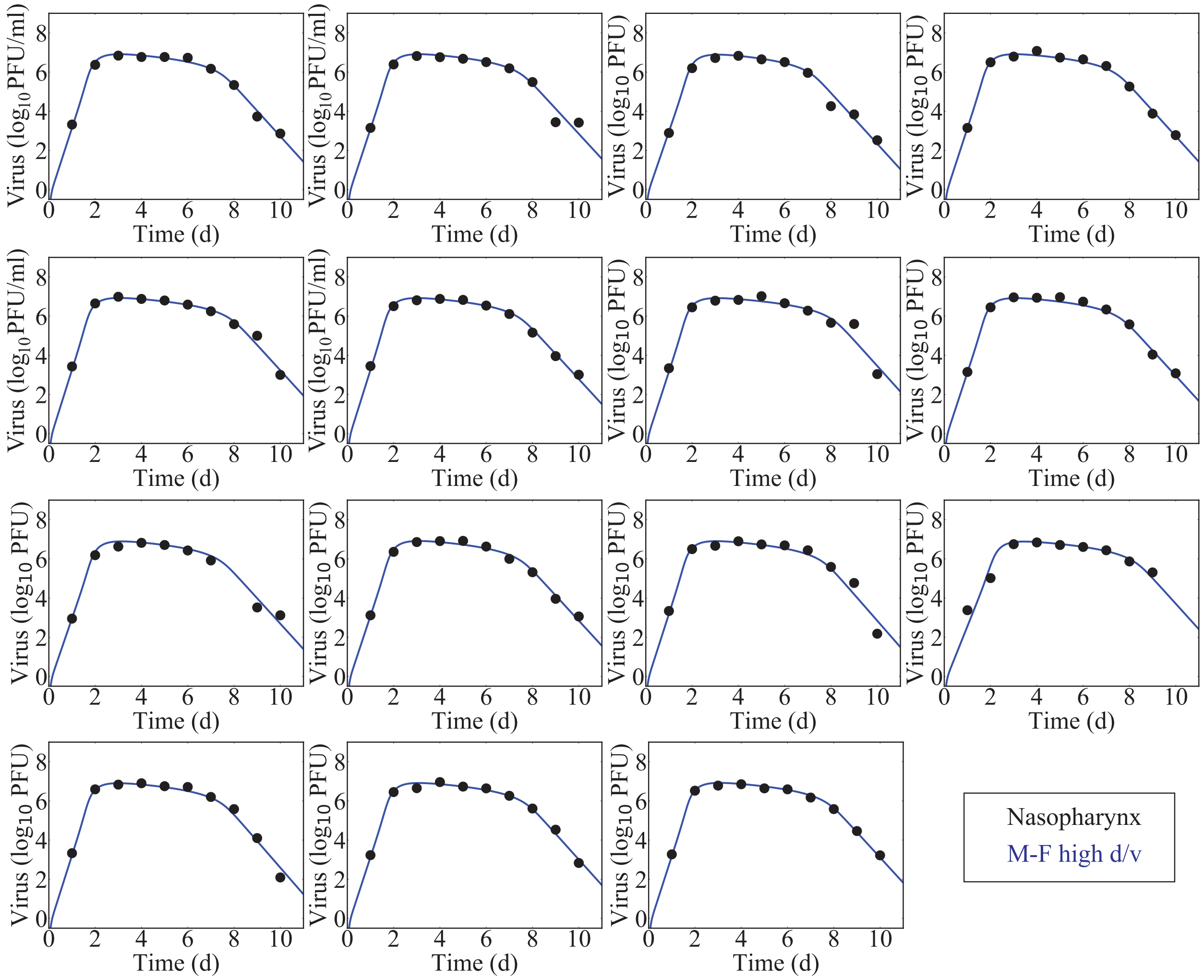

Figure T: Individual fits for the rSeV-luc(M-F*) at high d/v in the nasopharynx. Fit of the model (Eqs (3)-(6), Main Text) to the estimated viral loads from the nasopharynx of individual mice infected with rSeV-luc(M-F*) (black circles) at high d/v.

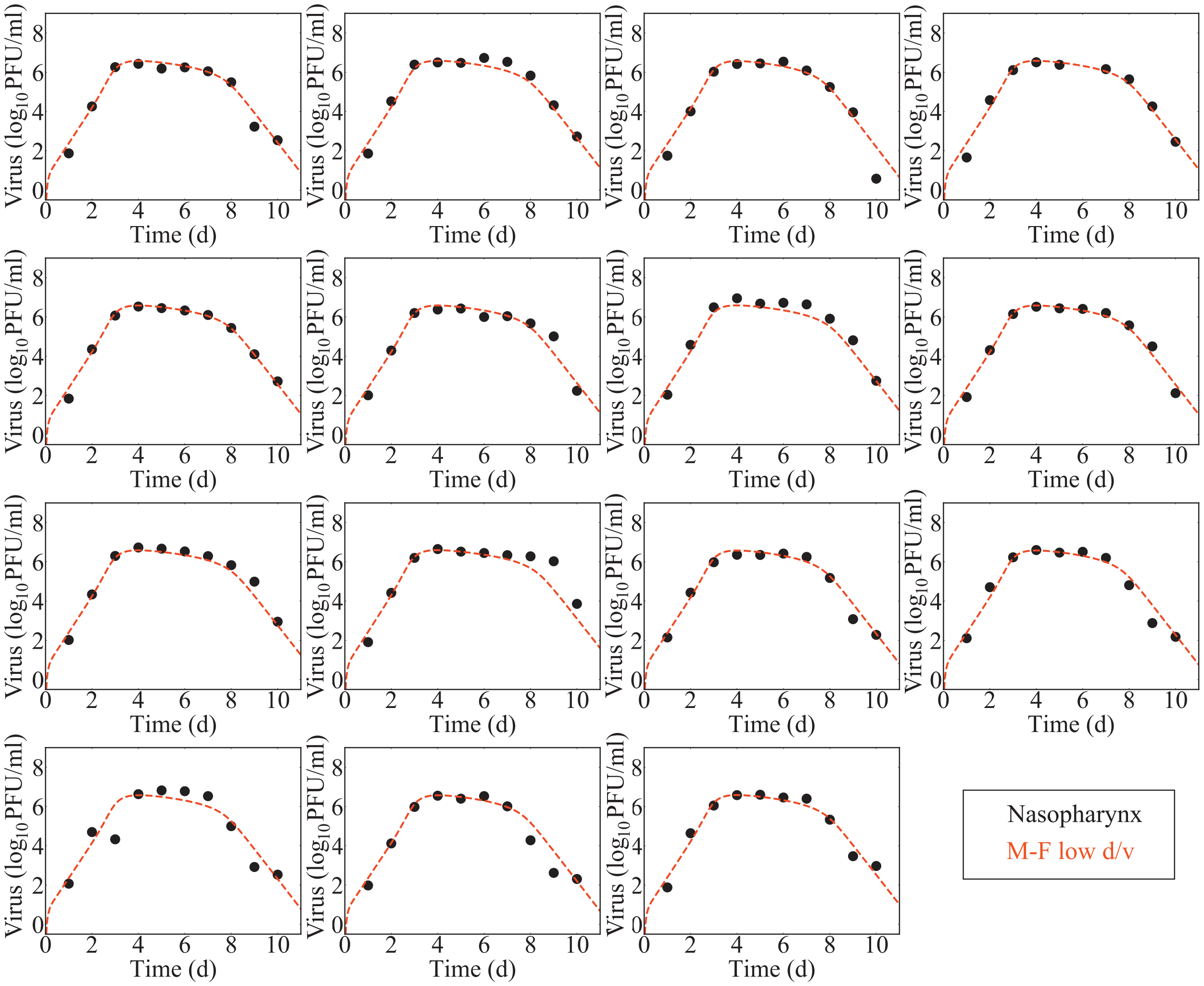

Figure U: Individual fits for the rSeV-luc(M-F*) at low d/v in the nasopharynx. Fit of the model (Eqs (3)-(6), Main Text) to the estimated viral loads from the nasopharynx of individual mice infected with rSeV-luc(M-F*) (black circles) at low d/v.

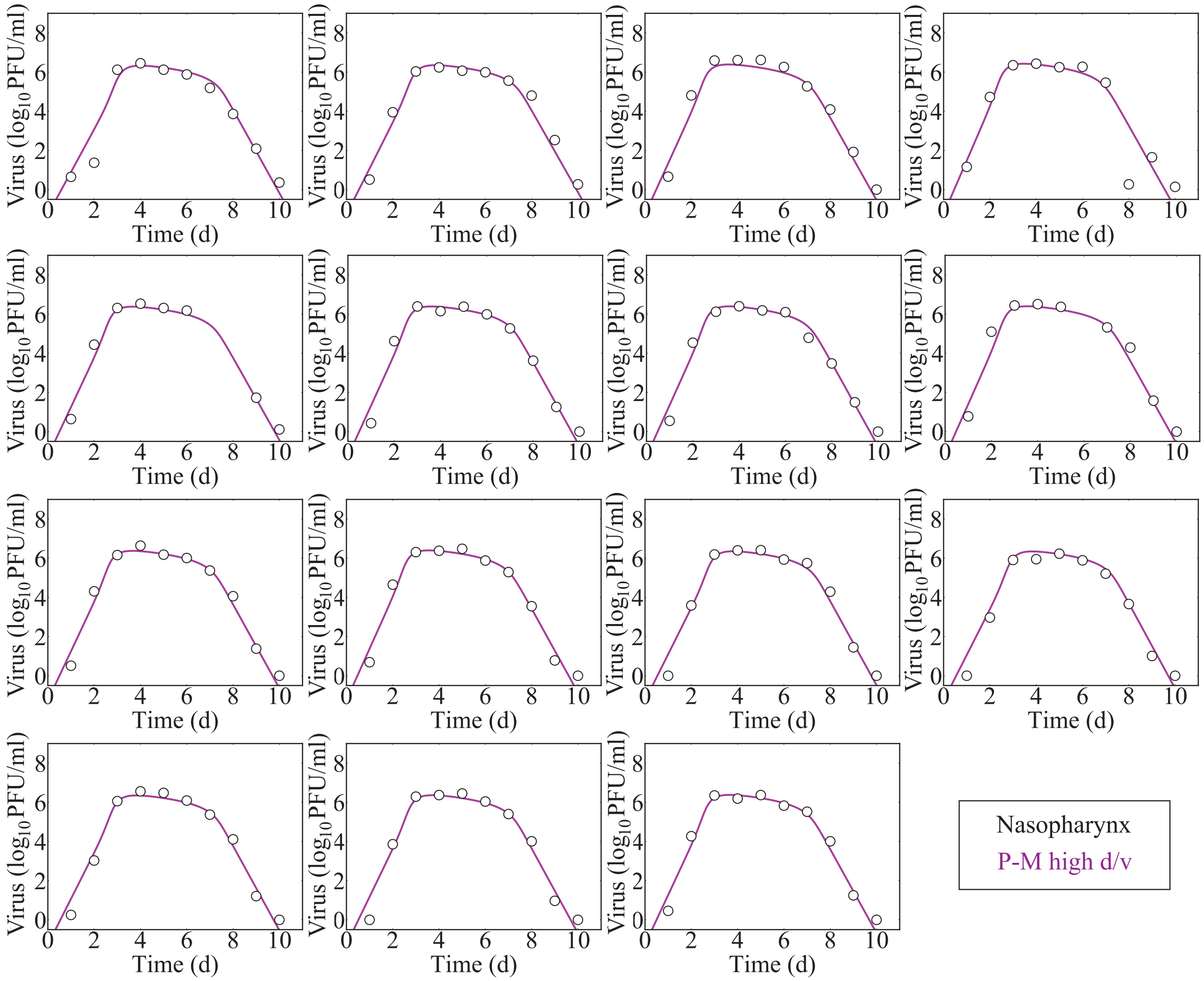

Figure V: Individual fits for the rSeV-luc(P-M) at high d/v in the nasopharynx. Fit of the model (Eqs (3)-(6), Main Text) to the estimated viral loads from the nasopharynx of individual mice infected with rSeV-luc(P-M) (white circles) at high d/v.

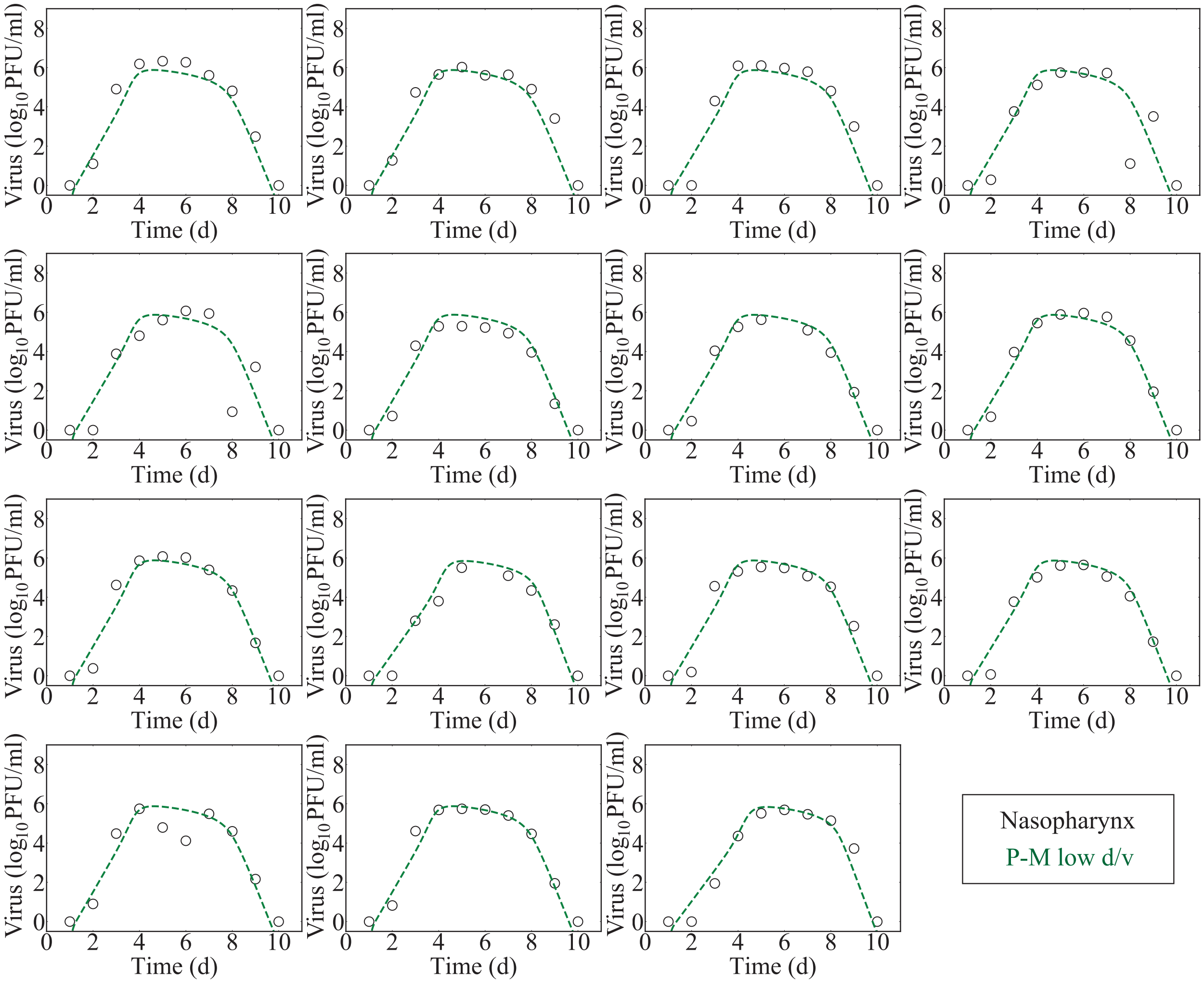

Figure W: Individual fits for the rSeV-luc(P-M) at low d/v in the nasopharynx. Fit of the model (Eqs (3)-(6), Main Text) to the estimated viral loads from the nasopharynx of individual mice infected with rSeV-luc(P-M) (white circles) at low d/v.
